# A two-dimensional axis estimation method for pickable canopy apples based on YOLO cascade network

**DOI:** 10.3389/fpls.2026.1904059

**Published:** 2026-08-20

**Authors:** Hongwei Cui, Meijia Yu, Shijie Jiang, Song Mei, Hao Ma, Zhengying Gu

**Affiliations:** 1 College of Agricultural Equipment Engineering, Henan University of Science and Technology, Luoyang, Henan, China; 2 Nanjing Institute of Agricultural Mechanization, Ministry of Agriculture and Rural Affairs, Nanjing, Jiangsu, China

**Keywords:** 2D axis estimation, apple harvesting, deep learning, instance segmentation, keypoint detection

## Abstract

**Introduction:**

In a complex orchard environment, canopy apples are obscured by various factors, making it hard for apple harvesting robots to accurately determine which apples can be directly harvested. Furthermore, the complex obstruction leads to difficulties in identifying keypoints on the apples and caculating the axis direction, directly affecting the robot’s determination of grasping positions.

**Methods:**

To solve these issues, a two-dimensional (2D) axis estimation method for pickable canopy apples based on a YOLO cascade network was proposed. Firstly, the study introduced SPDConv for lossless downsampling and adopts dynamic upsampling to improve segmentation boundary accuracy, constructing an instance segmentation network named the YOLO-SD model to select pickable apples according to different occlusion conditions and growth states. Secondly, the geometric center of the mask image was located using its minimum enclosing circle, and the region of interest was extracted through morphological dilation. Then, by integrating the RFAConv, SCSA attention, and MBConv modules, a keypoint detection network YOLO-RSM was constructed to extract keypoints of pickable apples. Finally, a 2D axis construction strategy was proposed, which adaptively constructs the growth axis based on the visibility of keypoints.

**Results:**

Experimental results show that the overall average accuracy mAP50 of the YOLO-SD model for apple segmentation reached 95.2%, and the parameter quantity was reduced to 2.47 M. The average accuracy of the YOLO-RSM model for keypoint detection has reached 90.3%, which is 2.4%, 2.6%, and 4.7% higher than that of the YOLOv8n, YOLO11n, and YOLO12n models respectively. The 2D axis estimation algorithm has an average axis angular error of 7.23° ± 16.73°, and an axis estimation accuracy of 92.68%.

**Discussion:**

The proposed method can achieve high-precision canopy apple segmentation, keypoint detection, and 2D axis estimation, thus offering technical support for the picking operations of apple harvesting robots.

## 1 Introduction

Apple is one of the most widely cultivated and economically valuable fruit in temperate regions worldwide. It also plays an essential part in the global fruit industry and agricultural trade. However, the current apple harvesting mode, which heavily relies on manual labor, is a typical labor-intensive operation. Inherently low production efficiency and rising labor costs of apple harvesting mode present a twofold challenge for industrial upgrading and sustainable development. In the global context where agricultural labor is increasingly scarce and labor costs are continuously rising, this traditional model can no longer meet the production demands of modern large-scale, intensive orchards (Zou et al., 2025). Therefore, developing robots that can automatically identify and picking apples with precision - to realize the mechanize and automate harvesting - has become an inevitable driver of industrial upgrading, cost reduction, and efficiency, and a major challenge in modern agricultural equipment.

During the development process of apple harvesting robot, precise identification of fruit targets is the primary step for automated harvesting (Wang et al., 2022). In early studies, apple fruits identification in orchards was mainly relied on image processing and machine learning, utilizing distinct visual features including fruit texture, color, and geometric shape (Wu et al., 2020; Yu et al., 2021). Those methods combined with threshold segmentation, edge detection, and morphological operations to separate fruit targets from the background and obstructions. However, these methods are easily effected by illumination variation, foliage occlusion, and fruit clustering, making them difficult to adjust to the complicated conditions of actual orchards. With the advancement of deep learning, extensive work has applied convolutional neural networks (CNNs) to orchard scenarios, enabling apple recognition and segmentation via bounding-box prediction (Hua et al., 2023). Although current deep learning methods have remarkably enhanced the recognition and segmentation accuracy (Wang and He, 2022; Jia et al., 2022), their large model size and high memory usage demand significant hardware resources from agricultural mobile platforms, hindering low-cost real-time deployment. Moreover, in unstructured orchards, apples are often severely obscured by branches and leaves or closely clustered with adjacent fruits (Zhou et al., 2022). Most studies mostly treat occlusion as a binary condition, lacking a refined semantic subdivision for different occlusions types (Li et al., 2024), and thus cannot adaptively adjust grasping strategies based on the real - time state of the apple fruit.

In addition, the orientation and position of the stem and calyx of an apple directly dictate the grasping strategy and path planning of the robotic manipulator. Precise detection of these key points facilitates fruit orientation estimation and allows the end-effector to achieve more accurate grasping. However, direct 3D axis recognition encounters difficulties in 3D data annotation and high computational power consumption during the training process. As a result, most research has shifted to detecting keypoints in 2D images and integrating 3D point cloud information to indirectly infer the axial information of fruits (Du et al., 2023; Zhao et al., 2025). Additionally, some researchers proposed a lightweight LPNet network specifically designed for apples, which employed an epidermis perception-based pose estimation method (Wang et al., 2026). Through the epidermis centralization annotation strategy and axial symmetry geometric constraints, they achieved a positioning accuracy of up to 93.6% and a real-time inference speed of 158 FPS, providing a solution for the precise estimation of apple picking points. Despite recent advances in keypoint detection accuracy (Geng et al., 2025), most existing models focus on whole fruit detection and largely overlook small scale targets such as fruit keypoints.

Although the accuracy of keypoint detection has improved in recent years (Geng et al., 2025), the existing research still has the following problems: (1) Most existing models focus on the detection and segmentation of apples in the entire scene, lacking the determination of whether the fruit can be directly picked by the current robot. Different types of occlusion and the spatial position of the fruit correspond to completely different picking strategies, while most methods simplify occlusion into a binary state, unable to provide the robot with refined target for picking. (2) Instance segmentation, keypoint detection, and pose estimation are usually studied as independent modules, lacking a unified framework to connect “which fruits can be picked” with “how to pick these fruits”. In summary, after completing the segmentation of tree crown fruits, how to determine which apples can be picked under complex lighting and background interference conditions, based on the posture information of the tree crown fruits, accurately locate the keypoints, calculate the appropriate picking direction and guide the robot to pick, remains an unsolved problem.

With the aim of overcoming the challenges encountered during the apple harvesting process, two objectives are set in this study: First, to construct a canopy fruit classification method based on growth state to determine which fruits are pickable by the robot. Second, to detect keypoints on pickable fruits and compute their orientation, thereby resolving the issues of fruit localization and picking direction for the harvesting robot. To achieve the above goals, the main contributions of this study are as follows: 1) A 2D axis estimation algorithm based on a two-level cascaded architecture is proposed. Firstly, the harvestable fruits are selected through instance segmentation, and then the keypoints of these harvestable fruits are predicted, finally the 2D axis of apples is calculated. 2) In the instance segmentation stage, a lightweight instance segmentation network YOLO-SD is proposed, which can perform fine semantic segmentation of apples with different occlusion types and growth states in complex orchards, and preliminarily determine the harvestable categories through semantic logic, serving as a basis for the subsequent robot’s picking sequence planning. 3) In the keypoint detection stage, a keypoint detection network YOLO-RSM is proposed to enhance the feature capture and recognition ability for small-scale keypoint targets (such as stems and calyxes).

## 2 Material and methodology

### 2.1 Data acquisition

The apple dataset was collected on August 2, 2024 from 9 am to 4 pm in an orchard in Luoning County, Luoyang City, Henan Province, with coordinates of 111°23′ E, 34°35′ N. The orchard is a low rootstock dense planting type orchard with a plant spacing of 2.5 m, a row spacing of 4m, and a tree age of six years. The main apple varieties planted are Fuji and Gala and the main research object of this paper is Fuji apples. A total of 1223 RGB images with a resolution of 3072 × 4096 were captured with the Huawei Mate60 mobile phone. The shooting methods included front light, back light, side light and cloudy days with no light, and the phone was positioned 1.2 m - 1.5 m away from the fruit tree. Taking into account the input of the YOLO model and the actual image resolution, the collected images were cropped to a resolution of 1024 × 1024. After removing blurry and images without apple instances, a total of 2194 images were retained.

### 2.2 Data pre-processing

#### 2.2.1 Data annotation

**Figure 1:**
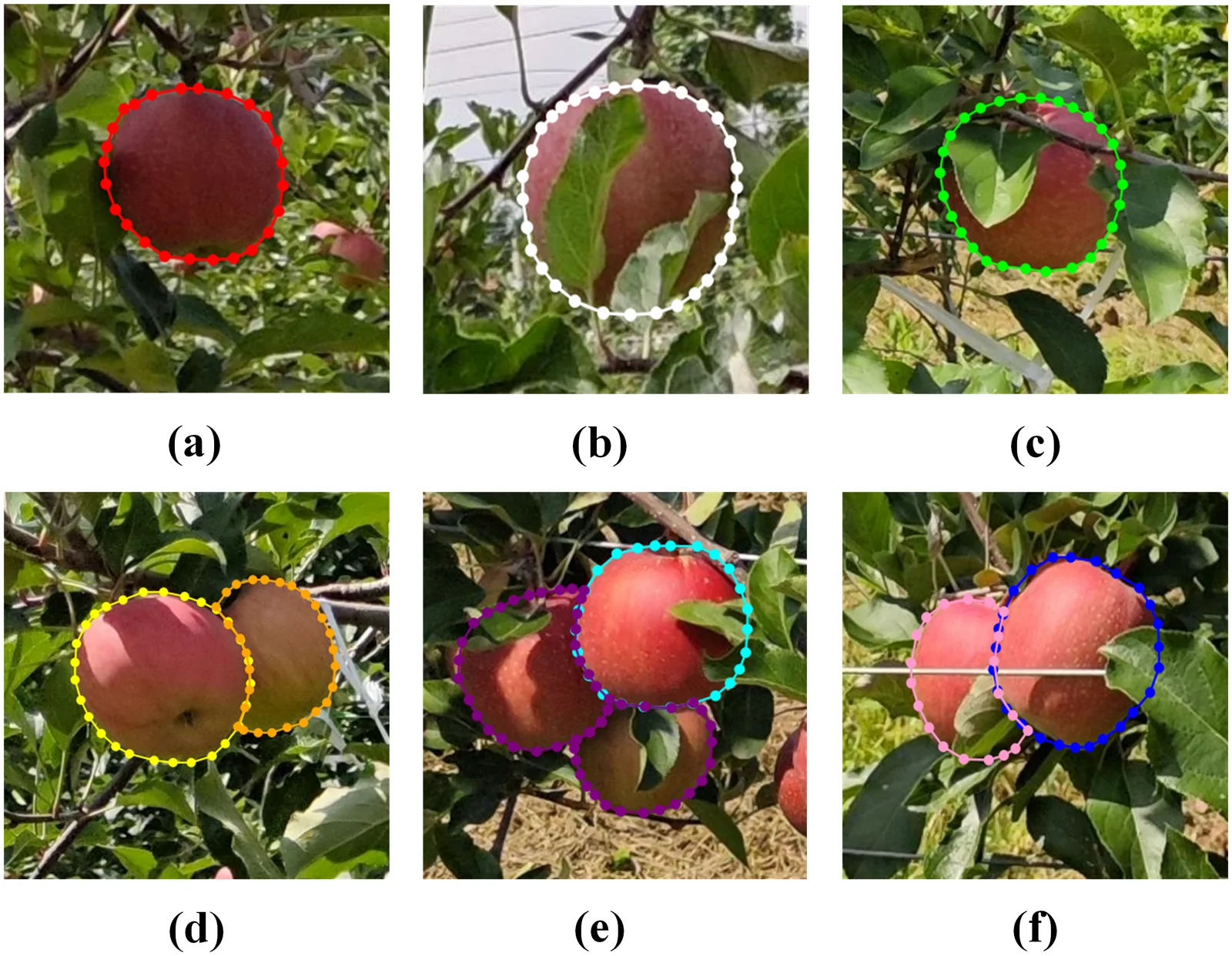
Examples of apple classification annotation. **(a)**single-apple-none, **(b)**single-apple-leaf, **(c)**single-apple-other, **(d)**The yellow box represents ‘multiple-front-none’, the orange box represents ‘multiple-back-none’, **(e)**The cyan box represents ‘multiple-front-leaf’, the purple boxes represent ‘multiple-back-leaf’, **(f)**The blue box represents ‘multiple-front-other’, the pink box represents ‘multiple-back-other’.

The apple instances were manually annotated by the Labelme software. In this process, polygon contours were used to outline each apple. Meanwhile, the samples were classified into the following nine categories according to their growth status and occlusion conditions: (1) single-apple-none (Single apples grow without any obstruction), (2)single-apple-leaf (Single apples grow with leaf obstruction), (3)single-apple-other (Single apples grow with other obstructions), (4)multiple-front-none (Apples grow at the front part of clustered fruits without obstruction), (5)mutiple-front-leaf (Apples grow at the front part of clustered fruits with leaf obstruction), (6)mutiple-front-other (Apples grow at the front part of clustered fruits with other obstructions), (7)mutiple-back-none (Apples grow at the back of clustered fruits without obstruction), (8)mutiple-back-leaf (Apples grow at the back of clustered fruits with leaf obstruction), (9)mutiple-back-other (Apples grow at the back of clustered fruits with other obstructions). Among them, other obstructions include those caused by non-harvestable objects such as tree branches. From the perspective of whether or not an apple can be harvested, the categories (1), (2), (4) and (5) were determined as harvestable objects. During actual picking, interference caused by leaf obstructions can be avoided by designing a reasonable mechanical claw structure and adopting a picking method. For example, an air-powered device can be integrated into the end effector, and when approaching the target, it can actively blow away the interfering leaves through a directional airflow or use a flexible adaptive mechanical claw to wrap the fruit in a circumferential manner along the axial direction and adopt a picking method of axial rotation to break the fruit stalk. Even if there are leaf obstructions, as long as the end effector can come into contact with the surface of the fruit and establish a stable grip, the picking can be completed through rotational torque. While branches and other objects belong to the category of rigid hard barriers. These two types have fundamentally different approaches in path planning and grasping strategies, so they need to be handled separately. This classification method ensures that each category corresponds to a clear picking strategy, avoiding redundant classification. The examples of the nine-category annotation for apples are shown in **Figure 1**.

The formulation of these nine types of standards is based on the following considerations: Firstly, the distinction between “front” apples and “back” apples is derived from the spatial front-back occlusion relationship between the fruits in the fruit cluster. We stipulate that the apples in a multi-fruit fruit cluster that are located in the front and not blocked by other apples are labeled as “front” apples, while the apples located in the rear and blocked by the front apples are labeled as “back” apples. The distinction between “single”, “multiple-front”, and “multiple-back” is because single fruits and the fruits in front of the cluster can be picked first, while the fruits at the back of the cluster need to handle the front obstruction or plan a path for avoiding obstacles in advance. These three have essential operational strategy differences. Secondly, the classification of the three types of occlusions is as follows: leaves belong to soft occlusion, while “other” obstructions such as branches belong to rigid hard occlusion. These two have fundamental differences in path planning and grasping strategies, so they need to be handled separately. This classification method ensures that each type corresponds to a clear picking strategy, avoiding redundant classification.

**Figure 2:**
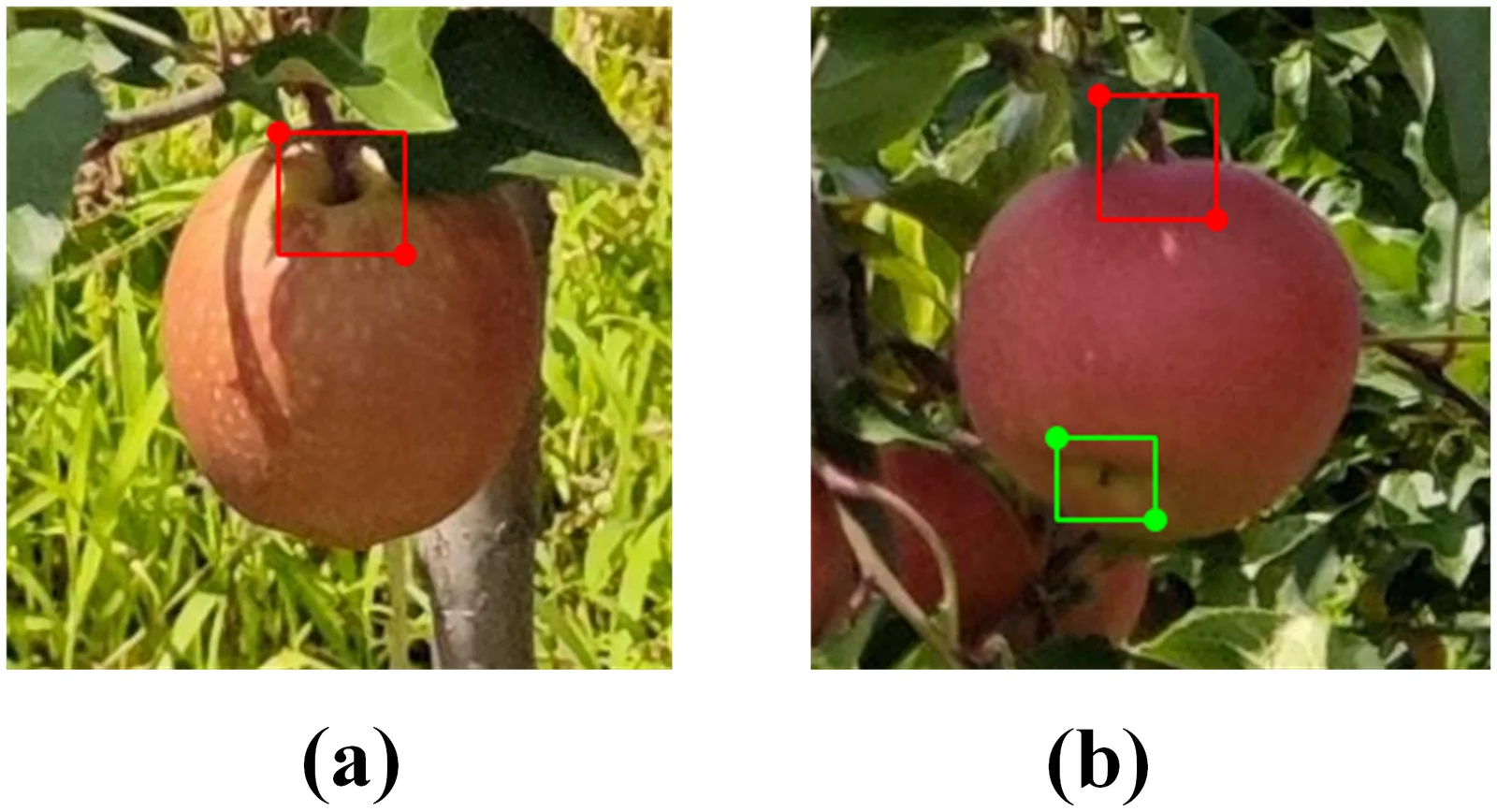
Examples of keypoint annotation. **(a)** The natural connection point between the stem and the fruit is visible. **(b)** The natural connection point between the stem and the fruit is invisible.

To extract the regional features of the stem and calyx keypoints, rectangular boxes were used to label the keypoints. The specific annotation example is shown in **Figure 2**. For the calyx, due to its prominent morphological features and easy identification, its location was directly delineated. For the stem, if the natural connection point between the stem and the fruit can be clearly observed, then the natural connection point between the stem and fruit was taken as the stem keypoint (**Figure 2a**). If the natural connection point between the stem and fruit cannot be seen due to the posture of apple, the connection point between the edge of the apple and the fruit stem was taken as the stem keypoint (**Figure 2b**). It is ensured that the keypoint is located at the center of the rectangular box, so the network is able to precisely learn the morphological features as well as spatial distribution of the stem and calyx around this center, strengthening the model’s perception and discrimination capability of subtle structural features. The center point of the final predicted rectangular box is the coordinate of the keypoint.

#### 2.2.2 Data augmentation

To enrich the diversity of data and enhance the generalization ability of the model, methods such as randomly adding Gaussian or salt-and-pepper noise, random translation, random mirroring and flipping, brightness adjustment and random rotation were randomly combined to expand the dataset. During the expansion process, the images and their corresponding annotation files underwent synchronous geometric transformation, and invalid annotations that completely exceeded the boundaries of the images after transformation were automatically filtered out. The dataset is as follows:

(1) Dataset for instance segmentation: in the original dataset, the samples of the ‘multiple-front-other’ and ‘multiple-back-none’ categories only have 664 and 442 respectively, while the sample numbers of the ‘single-apple-leaf’ and ‘single-apple-other’ categories are 2352 and 1905 respectively. The difference in the number of category samples is quite significant. To alleviate the problem of class imbalance, first, images that only contained categories with large amounts of samples were removed from the original dataset, reducing the total number of images from 2194 to 1654. On this basis, a class-balanced enhancement strategy was adopted. For images containing categories with fewer samples, perform double amplification; For other images, only single amplification is performed. After the balance and enhancement processing, the training set, validation set and test set were divided in a ratio of 7:2:1. Among them, the training set contains 3928 images, the validation set contains 982 images, and the test set has 432 images. **Table 1** presents the number of apple instances in each category before and after the data augmentation process.

**Table 1:** Number of apple instances before and after data augmentation.

Category	Before data augmentation	After data augmentation
all	10748	27012
single-apple-none	930	2848
single-apple-leaf	2352	4680
single-apple-other	1905	3878
multiple-front-none	850	2874
multiple-front-leaf	1269	2836
multiple-front-other	661	2112
multiple-back-none	442	1769
multiple-back-leaf	1149	2933
multiple-back-other	1190	3082

(2) Dataset for keypoint detection: after data augmentation, the keypoint dataset consists of a total of 7877 images, which are divided according to the ratio of 7:2:1, resulting in a training set of 5649 images, a validation set of 1413 images, and a test set of 715 images. The training set contains 8911 stems and 5730 calyxes. The validation set contains 2215 stems and 1544 calyxes. The test set contains 1044 stems and 778 calyxes.

### 2.3 2D axis estimation algorithm for harvestable canopy apples

**Figure 3:**
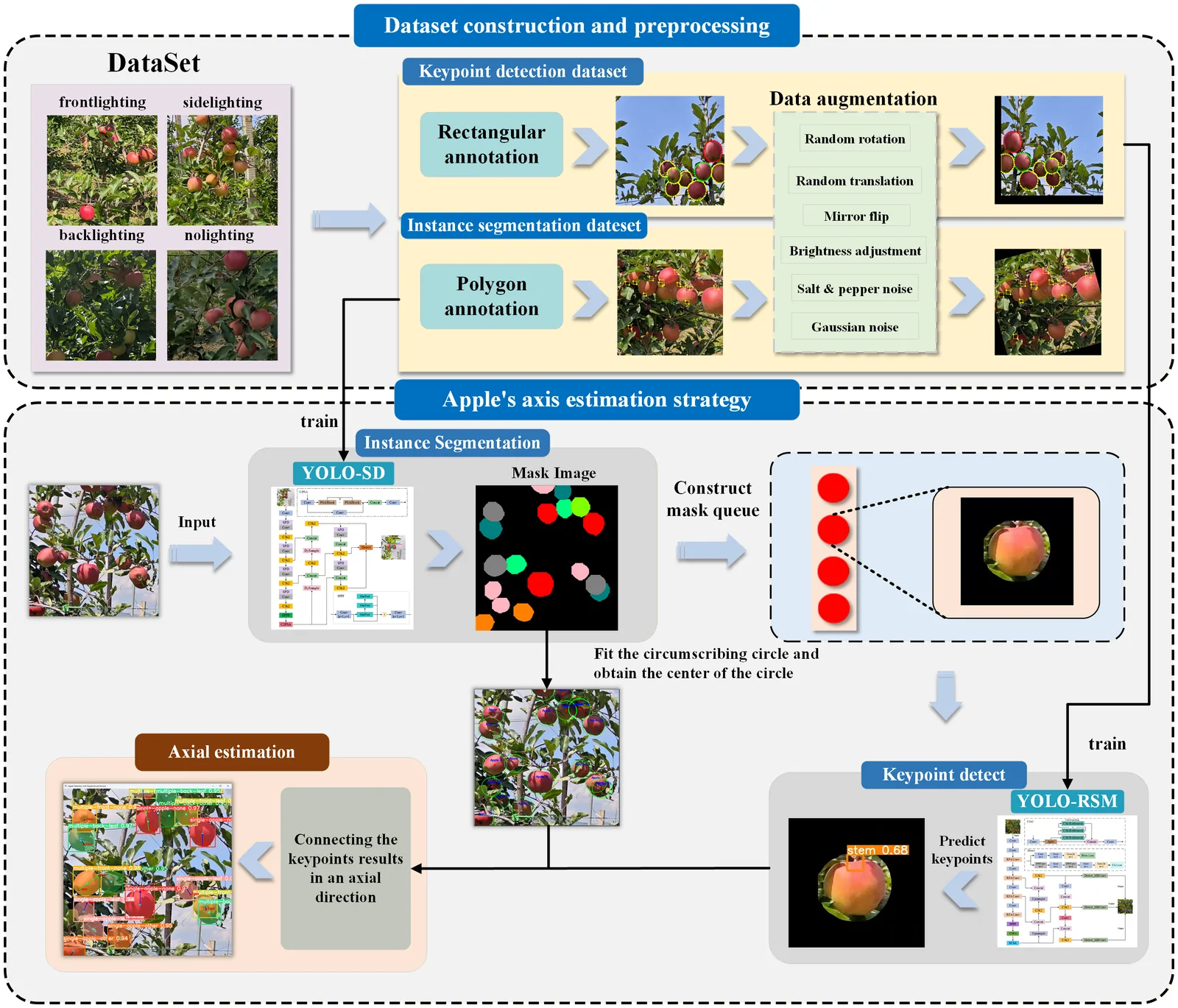
Apple 2D axis estimation algorithm.

This algorithm adopts a two-level cascaded network architecture. Firstly, the collected RGB images are input into the improved YOLO-SD instance segmentation network to achieve high-precision pixel level segmentation of apple fruits and extract instance masks for four types of harvestable apples. For each binary mask obtained, the algorithm performs the following operations in parallel: On the one hand, by extracting the maximum contour of the mask and applying the minimum surrounding circle algorithm for fitting, the 2D geometric center coordinates of the fruit are accurately calculated; on the other hand, to capture the characteristics of the stem or calyx adjacent to the edge of the fruit, the algorithm adopts morphological dilation operation, using elliptical structural elements to expand the original mask boundary outward by pixels. When determining the extension scale, if the extension range is too large, it is easy to introduce interference features from adjacent fruits, leading to false positives in the model; if the expansion range is insufficient, it will be difficult to fully capture the local contextual information required for keypoints, thereby weakening the feature representation ability. Hence, the expansion amount is ultimately set to 15 pixels in this paper. Subsequently, the extended mask is mapped back to the original image to extract ROI regions containing edge texture and keypoint information. Next, in order to accurately match individual apples and their stem and calyx keypoints, the algorithm will construct an apple mask queue, input the masks of all harvestable apple categories into the queue, and sequentially place individual masks on a pure black background to input into the keypoint detection network YOLO-RSM. After predicting the coordinates of the stem and calyx, the algorithm constructs an axis vector based on the visibility of keypoints and their positional relationship with the center point, achieving a complete reconstruction of the 2D growth axis of apples. In the actual picking operation, when the apples located in the preceding position are picked, the apples originally classified as the following category are exposed due to the elimination of obstruction, and automatically switch to the preceding state. The system dynamically incorporates it into the 2D axis estimation queue through real-time status updates, thereby achieving continuous recognition and adaptive adjustment of picking targets. The complete flow chart of the apple 2D axis estimation method is presented in **Figure 3**.

### 2.4 Canopy apple instance segmentation algorithm

#### 2.4.1 YOLO-SD model structure

**Figure 4:**
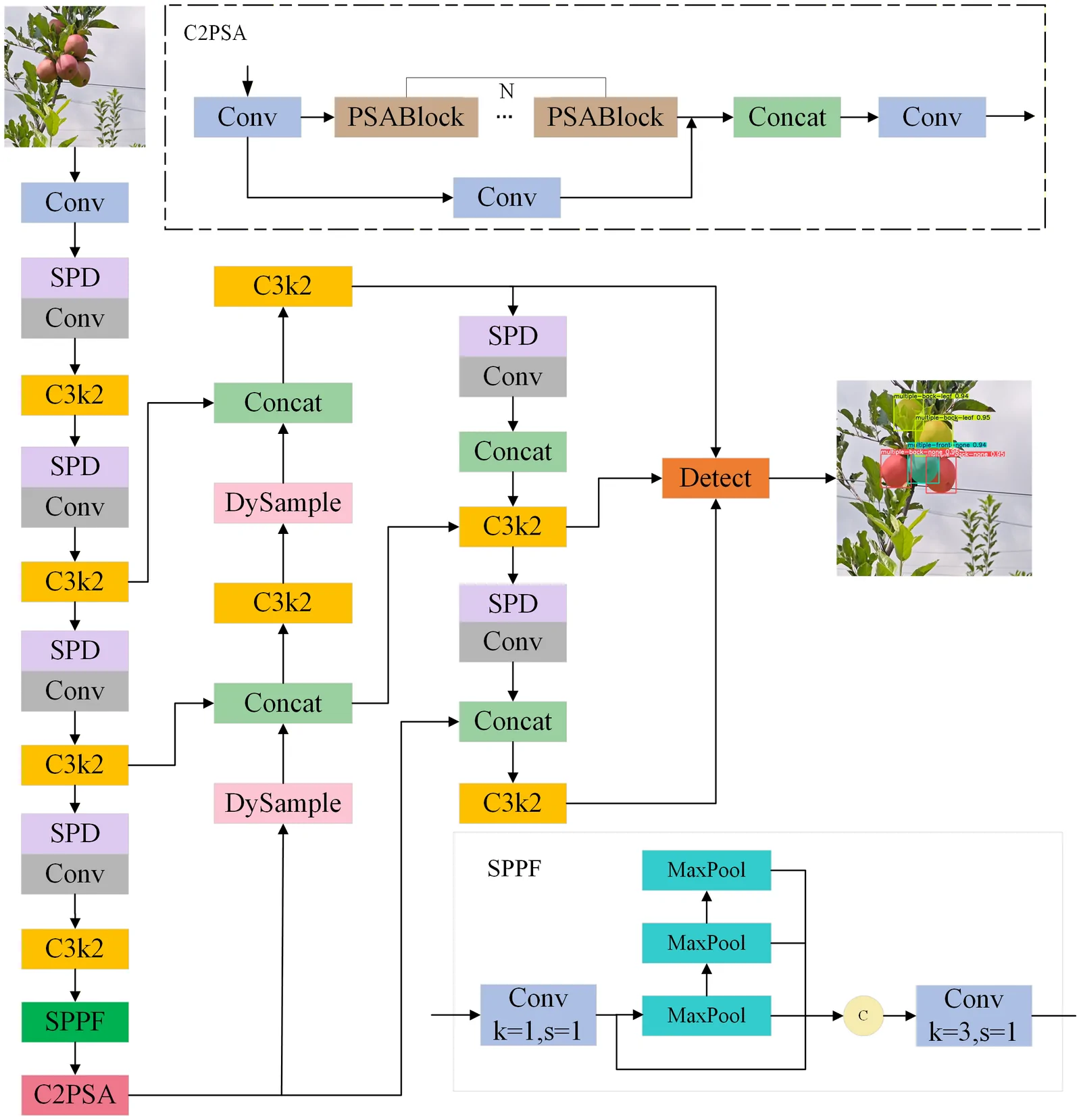
YOLO-SD model structure diagram.

To enhance the apple instance segmentation accuracy in complicated scenes, a lightweight YOLO-SD model based on YOLO11n as the baseline model is constructed. In the backbone network, SPDConv convolution is introduced to replace the standard convolution, and through spatial-to-depth transformation followed by non-step convolution, downsampling without information loss is achieved. In the upsampling stage, dynamic upsampling is used instead of the fixed interpolation method, and content-adaptive feature reconstruction is realized through learnable offsets, generating more accurate segmentation boundaries with extremely low computational cost. The model structure diagram is presented in **Figure 4**.

#### 2.4.2 SPDConv module

**Figure 5:**
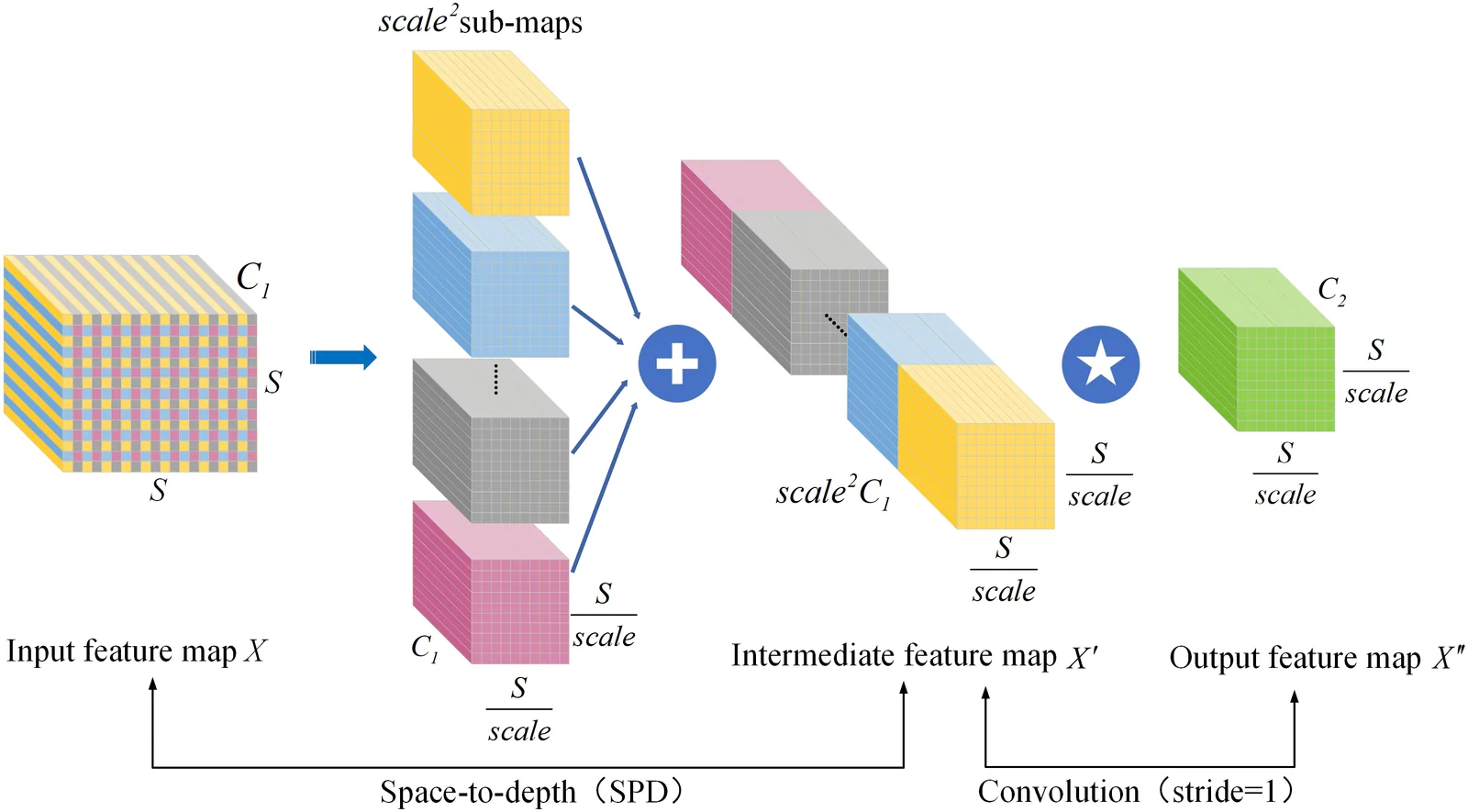
SPDConv module.

YOLO11n model uses stride convolution or pooling layers for feature map downsampling, which irreversibly loses key spatial information of apple fruits, especially for severely occluded apples. This information loss directly leads to blurred classification and inaccurate segmentation boundaries. SPDConv (Sunkara and Luo, 2022) achieves significant lightweight advantages by means of its unique operation of spatial-to-depth transformation followed by non-stride convolution, while also achieving downsampling without information loss: Firstly, as a parameter-free pixel recombination operation, the SPD layer does not introduce any additional computational weights. According to **Figure 5**, it splits the input feature map *X* (with dimensions *S × S × C_1_*) into scale^2^ subgraphs according to the scale factor *scale*. Each sub-image has dimensions $\frac{\text{S}}{\text{scale}}\times \frac{\text{S}}{\text{scale}}\times {\text{C}}_{1}$. Then, these subgraphs are merged to form the intermediate feature map *X′*, with dimensions $\frac{\text{S}}{\text{scale}}\times \frac{\text{S}}{\text{scale}}\times {\text{scale}}^{2}\cdot {\text{C}}_{1}$. This process preserves all pixel-level information and achieves spatial downsampling merely through rearrangement. Afterwards, the non-stride convolutional layer (with a stride of 1 and an output channel of *C_2_*) operates on *X′* to obtain the final output feature map *X′′*, with dimensions $\frac{\text{S}}{\text{scale}}\times \frac{\text{S}}{\text{scale}}\times {\text{scale}}^{2}\cdot {\text{C}}_{1}$. This operation utilizes learnable parameters to compress the channel redundancy caused by SPD transformation, and effectively extracts the features of input images, avoiding the asymmetric sampling and detail loss caused by traditional step length convolution. It improves the model’s discriminative power for subtle differences between multiple categories and the accuracy of segmentation masks, meanwhile decreasing the computational complexity and number of parameters.

#### 2.4.3 DySample module

**Figure 6:**
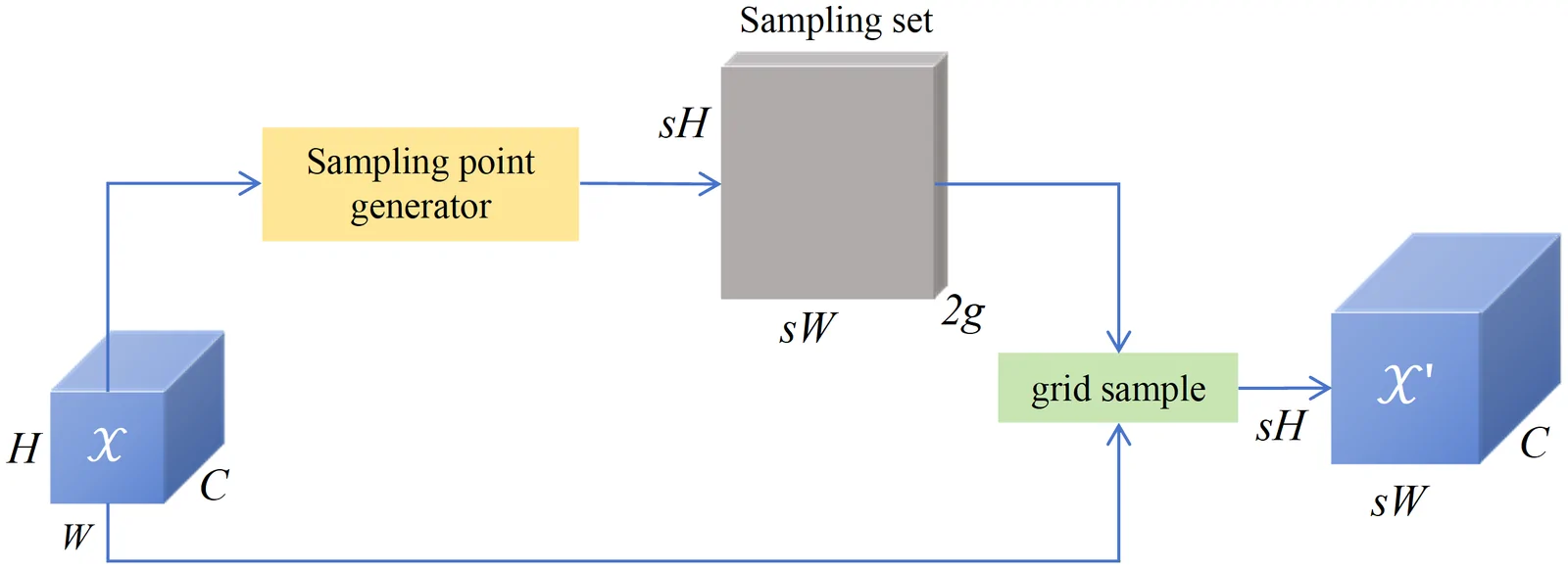
DySample module.

The standard upsampling method uses a fixed interpolation kernel, which makes it difficult to accurately restore the semantic boundaries of the overlapping fruits and the fruits under complex occlusion in the low-resolution feature reconstruction. In this study, the DySample (Liu et al., 2023) upsampler was integrated into the YOLO11n apple fruit instance segmentation model, replacing the original standard upsampling strategy. Through a dynamic content-aware point sampling mechanism, more detailed feature reconstruction was achieved with extremely low computational overhead. Specifically, the DySample module dynamically adjusts the sampling position of each upsampling point through learnable offsets, and its principle is as follows: For the input feature map $X\in {\text{R}}^{C\times H\times W}$, DySample module first feeds it into a sampling point generator with a built-in upsampling scale factor *s* (**Figure 6**). The generator generates a spatial offset $O\in {\text{R}}^{2\times sH\times sW}$ through a lightweight linear layer, and adds it to the initial sampling grid *G* to obtain the dynamic sampling set Sampling Set (*S*) (Equation 1):

(1) S=G+O

Then, the continuous feature maps are resampled using the built-in ‘grid_sample’ function of PyTorch, generating a high-resolution output (Equation 2):

(2) $$X^{\prime }=grid\_sample\left(X,S\right)$$

During this process, the DySample module ensures the stability of sampling and its adaptability to different semantic features through bilinear initialization, dynamic range factor constraints, and group sampling mechanism. Meanwhile, it relies solely on highly optimized native functions of PyTorch, without the need for custom CUDA kernels, significantly reducing parameters and computational overhead while keeping inference speed close to bilinear interpolation.

### 2.5 Canopy apple keypoint detection algorithm

#### 2.5.1 YOLO-RSM model structure

**Figure 7:**
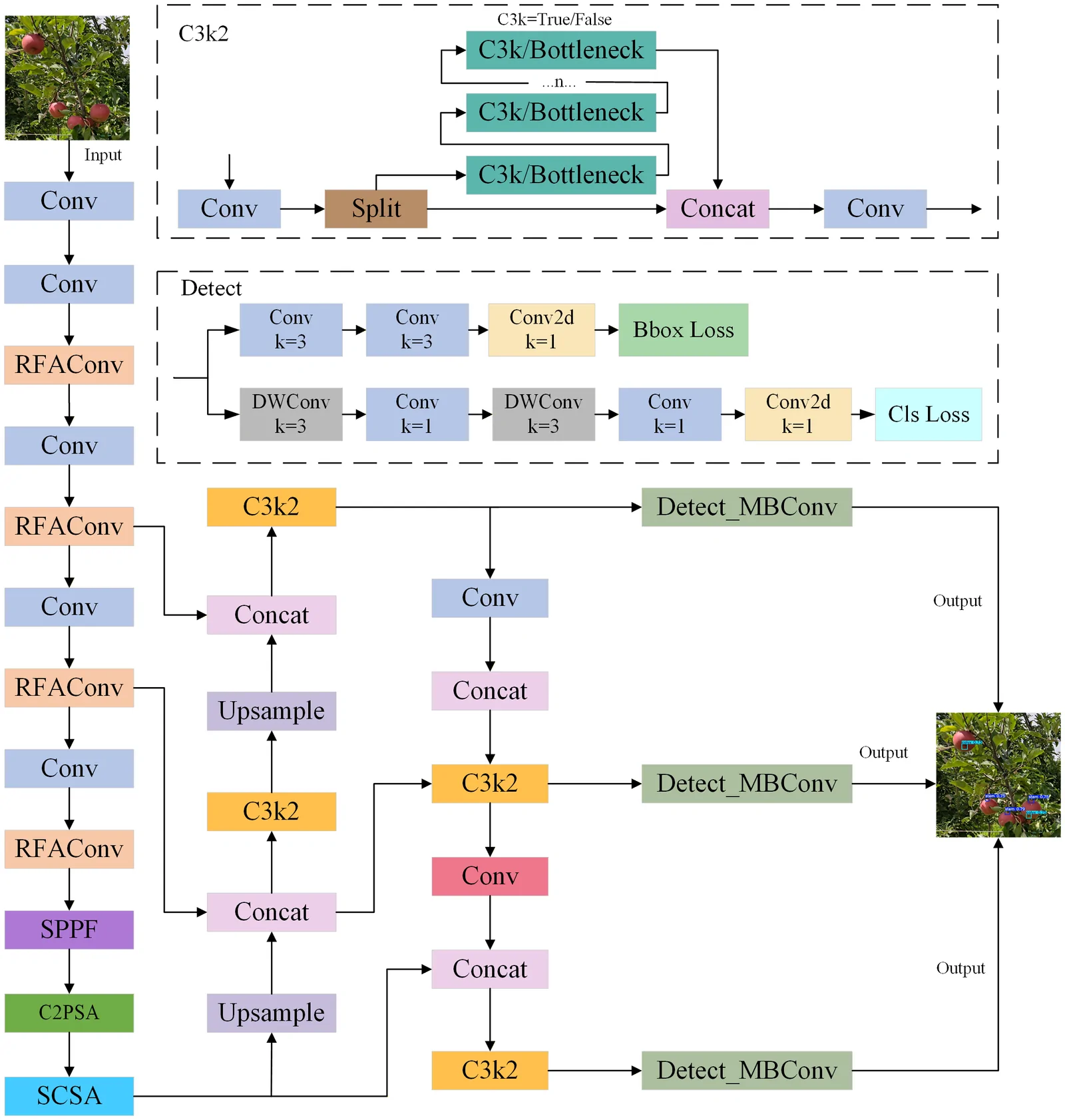
Network architecture of the YOLO-RSM.

The current series of YOLO-based algorithms have demonstrated strong performance in object detection tasks (Fan et al., 2022), but in complex orchard environments, small-scale keypoint targets are often occluded and interfered by lighting conditions, resulting in weak visual features. Moreover, the limited receptive field makes it difficult to capture the contextual semantics of keypoints, leading to insufficient positioning accuracy. To solve this problem, we took YOLO v11 as the baseline model and designed a YOLO-RSM architecture (**Figure 7**). This architecture introduces RFAConv module and SCSA attention mechanism into the backbone network, and integrates MBConv module in the detection head to enhance the model’s ability to recognize small-scale targets such as stems and calyxes of apple fruits.

#### 2.5.2 RFAConv module

**Figure 8:**
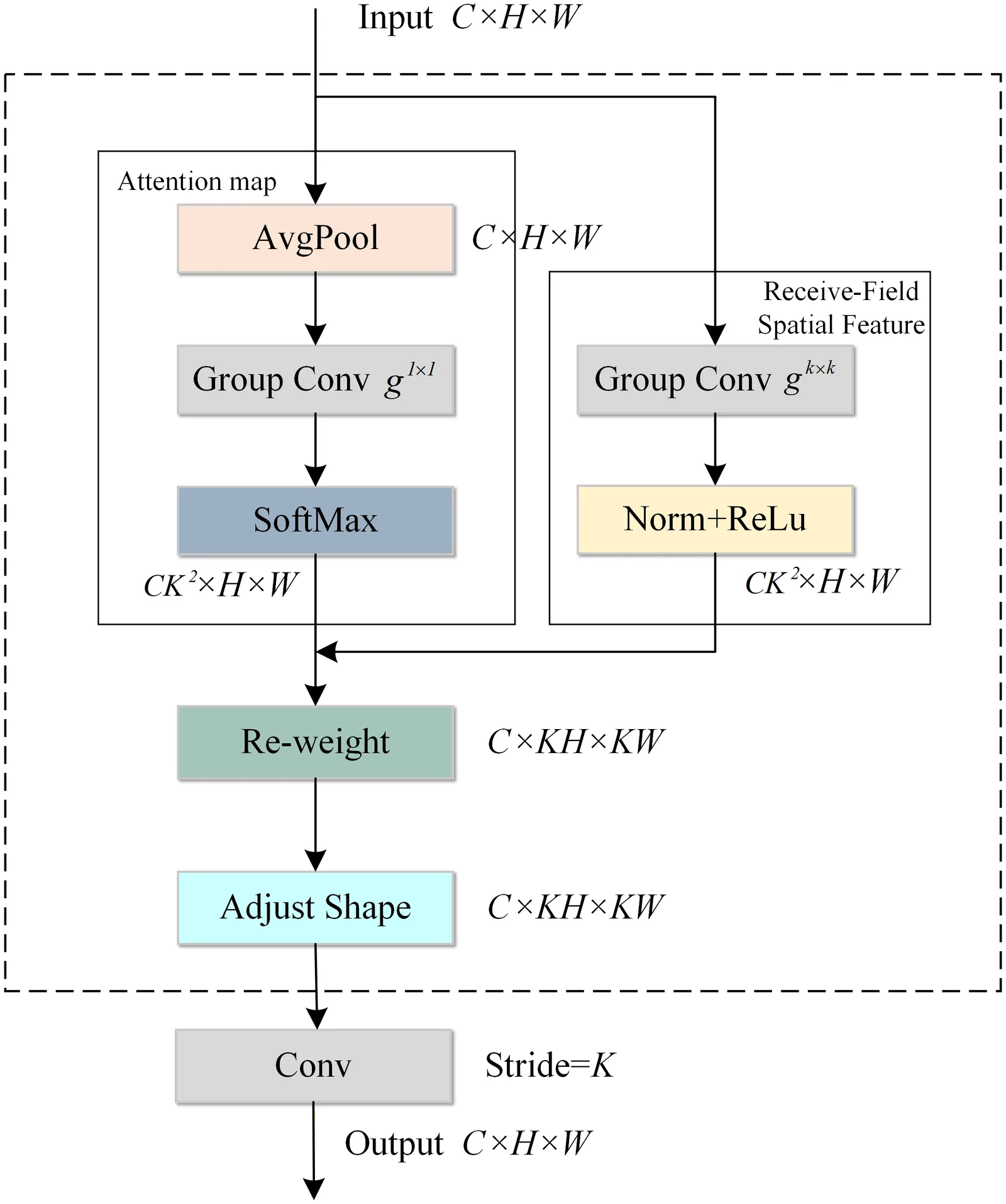
The structure of RFAConv module.

In standard convolution, the convolution kernel shares the same set of parameters at all spatial positions of the input feature map X. This mechanism prevents the model from adaptively extracting features based on the local context environment of the stem and calyx. In contrast, the RFAConv (Zhang et al., 2023) module reconstructs standard convolution operations through receptive field attention (RFA), completely solving the challenge that traditional convolution kernels are insensitive to local spatial differences caused by parameter sharing during the sliding window process. Specifically, the RFAConv module converts input features into non overlapping receptive field spatial features *Frf* through efficient grouped convolution *g^k^*^×^*^k^*. Subsequently, it reshapes the model’s feature perception ability through a dual branch structure. On the one hand, global average pooling with zero parameter aggregates the information inside the receptive field, and then a very lightweight group convolution *g*^1×1^ is employed for feature interaction to generate the attention map *Frf*. On the other hand, a group convolution *g^k^*^×^*^k^* is applied to the receptive field spatial feature *Frf* for preliminary mapping, and the transformed features are normalized and nonlinearly activated (**Figure 8**). The core process can be expressed in Equation 3:

(3) $$F=Softmax(g^{1\times 1}(AvgPool(X)))\times ReLU(Norm(g^{k\times k}(X)))=Arf\times Frf$$

This mechanism assigns adaptive weights (Re-weights) to each receptive field region, making the network pay more attention to the local subtle features where stem and calyx keypoints are located, thereby enhancing sensitivity to small targets and occlusion situations.

#### 2.5.3 SCSA module

The stem and calyx of an apple account for a very small proportion of the overall pixel of the apple target. Their texture features are often blurry and similar to tree branches and other obstructions. With the number of YOLO network layers increasing, these subtle spatial information are prone to be lost. The core mechanism of Spatial and Channel Synergistic Attention module (SCSA) (Si et al., 2024) is composed of the Shareable Multi-Semantic Spatial Attention (SMSA) and the Progressive Channel-wise SelfAttention (PCSA) connected in series: The SMSA in SCSA can extract multi-level spatial information of apple shape, texture, and keypoint distribution, and maintain the independence of different semantic sub features through group normalization to avoid semantic interference; the PCSA method uses the spatial prior given by the SMSA to guide adaptive recalibration of channel features, enhancing the response to keypoint-related features. Meanwhile, through the self-attention mechanism between channels, it alleviates semantic differences caused by apple posture, occlusion, or uneven illumination, achieving the collaborative enhancement of spatial and channel information. Finally, the channel weights are generated through Sigmoid activation and weighted fused with the original spatial features, which improves the model’s perception of local details and global context and maintains its original efficient inference ability, thus achieving more accurate detection results in apple keypoint localization.

#### 2.5.4 MBConv module

**Figure 9:**
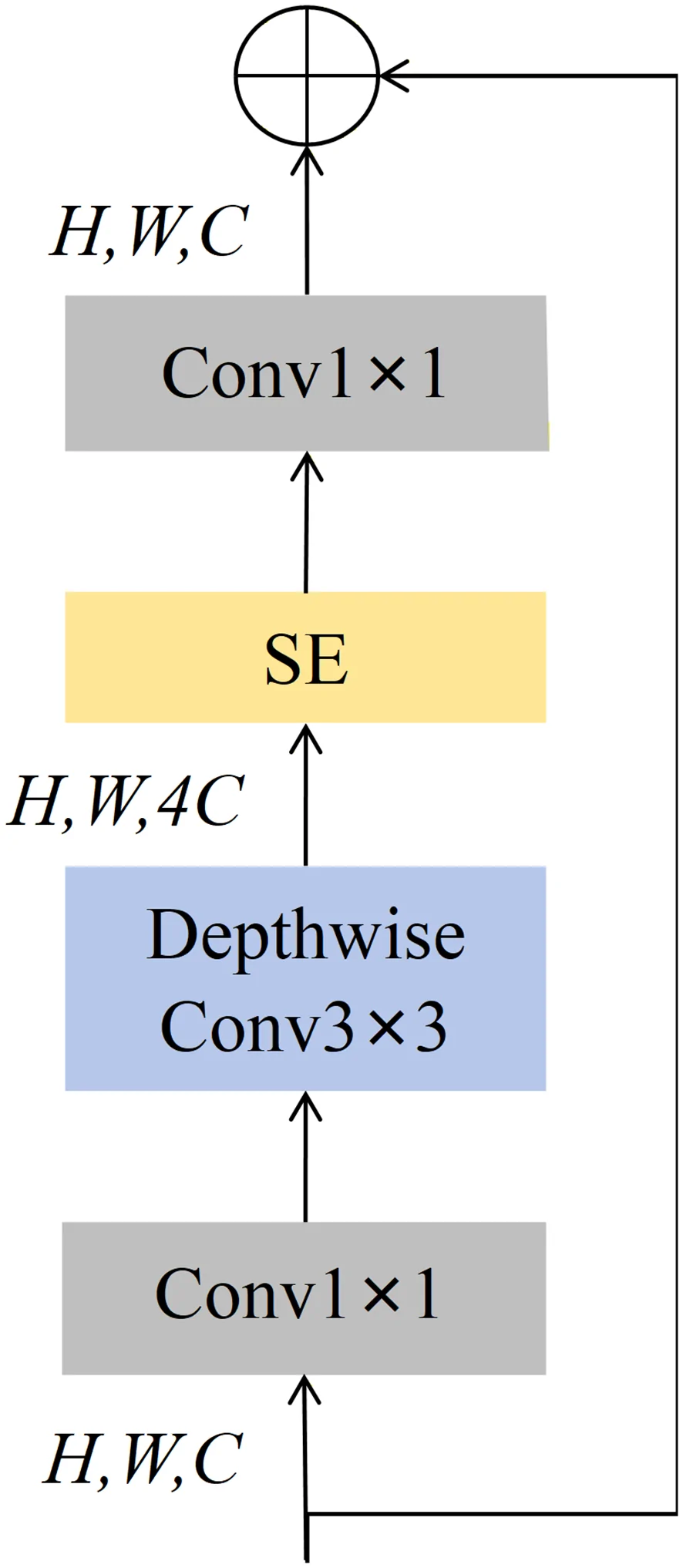
MBConv module.

The traditional detection head adopts a standard convolution architecture with high computational redundancy and lacks a channel attention mechanism, which leads to limited inference speed of the model and making it difficult to avoid the loss of subtle features caused by nonlinear transformations. In this paper, the Mobile Inverted Bottleneck module (MBConv) (Tan and Le, 2019) proposed by EfficientNet is merged into the detection head. This module introduces an inverted bottleneck structure, making the network capture more feature information with fewer parameters, thereby enhancing the discrimination ability of the detection head for complex and subtle features: Channel dimension is firstly expanded by a 1×1 convolution, after which a depthwise separable convolution is employed for spatial feature extraction. Finally, the channels are compressed back to the target dimension using another 1×1 convolution and the channel weights are adaptively recalibrated by combining the SE module, which greatly reduces the parameter count and computational costs. The depthwise separable convolution here consists of depthwise convolution and pointwise convolution (Balasundaram et al., 2023). Deep convolution is a convolution operation performed channel by channel, where each convolution kernel is only responsible for one input channel. It can efficiently extract spatial information of features without significantly increasing computational complexity. Pointwise convolution linearly weights and combines the feature maps output by deep convolution in the channel dimension. The two work together to effectively reduce the computational complexity. The Squeeze and Excitation module (SE) aggregates global contextual information through global average pooling and generates channel level attention weights. This enables the detection head to adaptively recalibrate the responses of each feature channel, enabling it to concentrate on the discriminative features representing the stem and calyx of apples from the complex background more accurately, and capture the fine spatial information of apple stems and calyxes more reliably. **Figure 9** presents the structure of MBConv.

### 2.6 Apple 2D axis estimation method based on keypoint visibility

After predicting the coordinates of the stem and calyx, the algorithm dynamically constructs an axis vector based on the visibility of keypoints. If both the stem and calyx keypoints were visible, a vector line pointing from the calyx to the stem was constructed as the 2D axis direction of the apple. If only a single keypoint was detected, we first extract the largest contour from the binary mask of the apple output by the YOLO-SD model, then obtain the center of the circle using the minimum enclosing circle fitting algorithm and use it as the geometric center of the apple. We construct vector lines pointing from the center to the stem (only the stem is visible) or from the calyx to the center (only the calyx is visible) as the 2D axis directions of the apple, thus achieving a complete reconstruction of the apple growth posture in 2D space. If both keypoints were not visible, it is difficult to accurately determine their growth posture. Therefore, they were filtered out and not considered for picking in this method.

## 3 Experiments and results

### 3.1 Evaluation metrics

To assess the performance of the model comprehensively, multiple evaluation metrics need to be taken into consideration. In this study, precision (P), recall (R), average precision (AP), mean average precision (mAP) (Jin et al., 2025), parameters, model size and GFLOPs were adopted as the evaluation metrics. The relevant formulas can be found in Equations 4–7. Among them, mAP was further evaluated as mAP50 and mAP50–95 to comprehensively assess the model’s performance under different positioning accuracy requirements. The metrics were computed using the following equations:

(4) $$Precision=\frac{TP}{TP+FP}$$

(5) $$Recall=\frac{TP}{TP+FN}$$

(6) $$AP=\int_{0}^{1}P(r)dr$$

(7) $$mAP=\frac{1}{N}\sum_{i=1}^{N}{AP}_{i}$$

Where, *TP* represents true positives, *FN* stands for false negatives, *FP* stands for false positives, *P*(*r*) represents the relationship between precision and recall; N represents the number of categories, AP_i_ represents the *AP* of the i-th category. The model size and parameters serve as indicators of the storage resources required by the model. The GFLOPs metric is employed to quantify the computational complexity associated with running the model.

To quantitatively assess the model’s prediction performance for apple growth posture, the Mean Angular Error (MAE) and Axis Estimation Accuracy (AEA) are used as evaluation indicators. MAE is used to measure the average value of the angle between the predicted axis vector *v_pred_* and the manually annotated true value vector *v_gt_*, reflecting the absolute regression accuracy of the algorithm, as shown in Equation 8.

(8) $$MAE=\frac{1}{N}\sum_{i=1}^{N}\theta_{i}$$

The calculation formula for the single sample angle error θ is presented in Equation 9:

(9) $$\theta =arccos\left(\frac{v_{pred}\cdot v_{gt}}{\left|v_{pred}\right|\times \left|v_{gt}\right|}\right)\times \frac{180}{\pi }$$

Where, the smaller the MAE value is, the higher the model’s regression accuracy for the posture.

Additionally, considering the certain grasping tolerance of the end effector of the robotic arm in actual picking operations, this paper introduces the index AEA to evaluate the algorithm’s engineering feasibility. The index represents the proportion of samples with angle errors within the preset threshold. Taking into account the optimal picking angle range of the end effector, the mechanical conditions for fruit stem separation, and the motion control accuracy of the robotic arm, this study takes τ = 15° as the preset threshold. The calculation formula is listed in Equation 10:

(10) $$AEA=\frac{N_{correct}}{N_{total}}\times 100\%$$

where N_correct_ stands for the total number of samples which meet the condition *θ* ≤ *τ*.

### 3.2 Experiment settings

**Figure 10:**
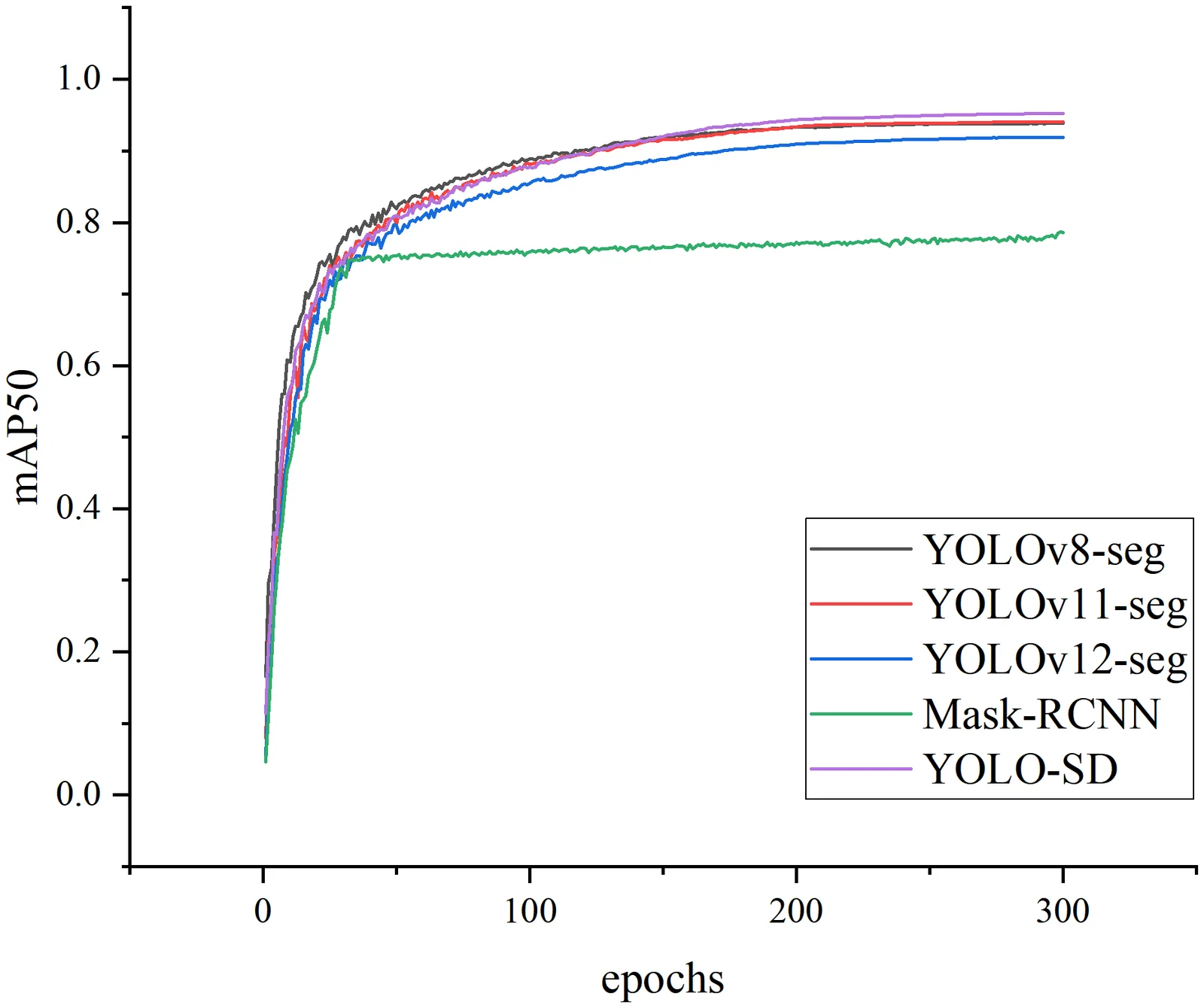
The mAP curve of different instance segmentation models.

**Figure 11:**
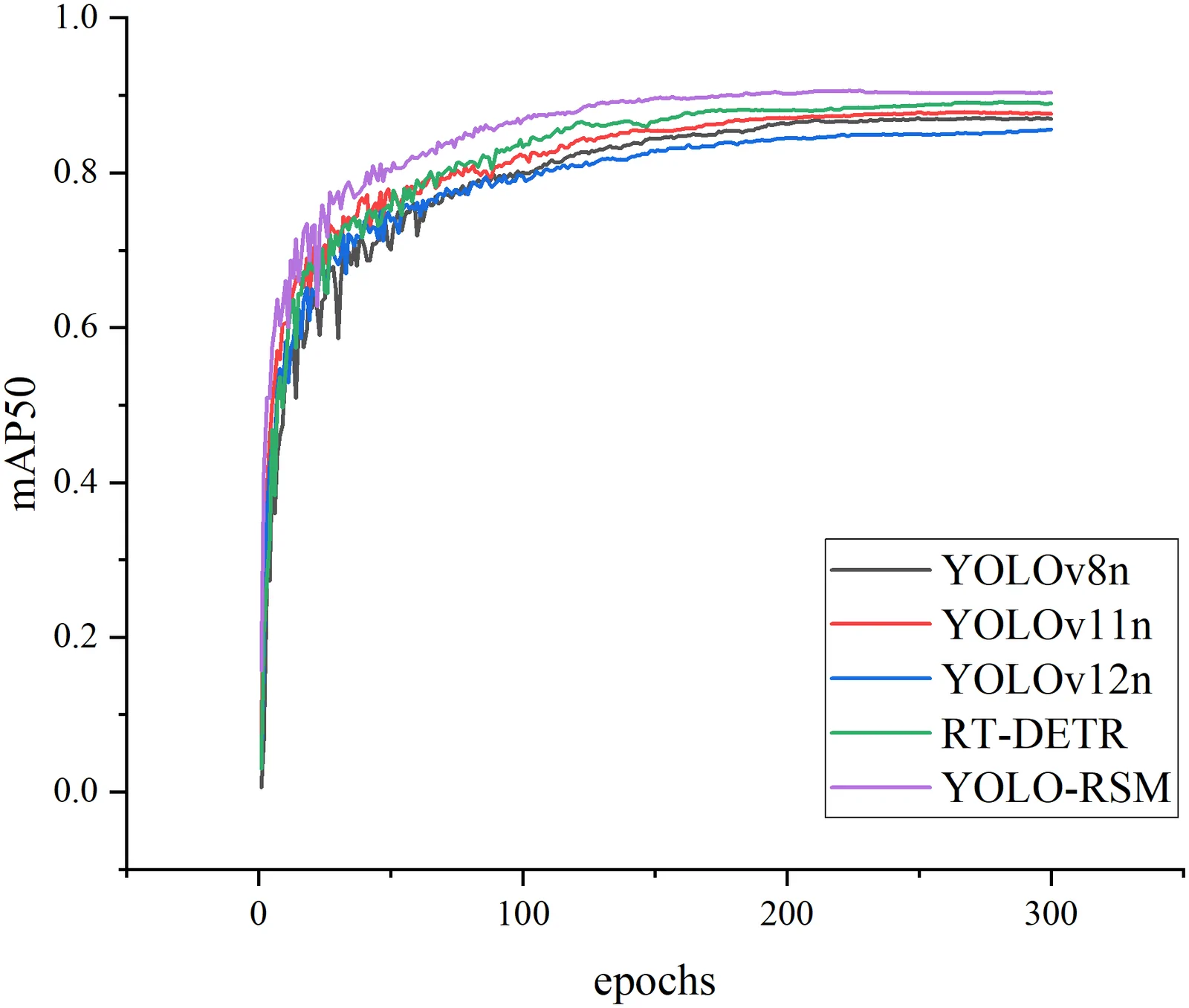
The mAP curve of different detection models.

The original YOLO11n was used as the baseline model in this study. To guarantee both consistency and reproducibility of the experiments, the whole model training and testing tasks were conducted on Ubuntu 22.04 operating system, with a hardware configuration consisting of an Intel Core i9-14900KF central processor and an NVIDIA GeForce RTX 4080 graphics processor, utilizing CUDA 12.4 as the GPU acceleration library. The deep learning framework employed was PyTorch 2.5.1, and the programming language used was Python 3.12. In terms of hyperparameter settings, considering the utilization rate of hardware resources and the memory limit, the batch size was uniformly configured as 16. The number of model training iterations as 300, which is consistent with the standard training protocol of the YOLO series, and it has been verified in subsequent experiments that this iteration count is sufficient for the model to converge fully on the dataset (**Figures 10**, **11**). To preserve the fine-grained features of small-scale keypoints and enable the model to capture richer texture information during downsampling, the network input size is set to 1024×1024. As for training strategy, the network was trained using the AdamW optimizer, with an initial learning rate of 0.001 and worker threads of 8. This optimizer decouples weight decay from the gradient update process, effectively mitigating overfitting and improving model stability compared to the standard Adam optimizer. All hyperparameters remain consistent across comparative experiments to ensure valid evaluation of architectural improvements.

### 3.3 Instance segmentation results and analysis of canopy apples

#### 3.3.1 Ablation experiment

In the ablation experiment of instance segmentation, this paper gradually introduced the SPD-Conv and DySample modules based on the original YOLO11 segmentation model to assess their individual and joint effects on model performance.

The results in **Table 2** suggest after introducing the DySample module, the model’s precision, recall, and mAP50 improved by 1.6%, 1.1%, and 1.0% respectively, while mAP50–95 increased by 1.3%. Meanwhile, the parameter quantity and computational load remained largely unchanged. This indicates that the module can effectively enhance feature reconstruction quality through a dynamic sampling mechanism without additional computational overhead, thereby improving segmentation accuracy. After introducing the SPD-Conv module, the model achieved a 1% improvement in mAP50 while reducing the parameter quantity to 2.46 M, GFLOPs to 9.3, and compressing the model size from 6.1 MB to 5.4 MB. This demonstrates the module’s effectiveness in lightweighting the model by optimizing the convolutional structure while maintaining detection performance. Ultimately, through the synergistic effect of both modules, the highest mAP50 of 95.2% was achieved. Compared to the original model, the parameter quantity, floating-point operations, and memory usage were reduced by 12.7%, 8.8%, and 11.5% respectively, significantly lowering computational and storage demands while improving performance, achieving a balanced optimization of accuracy and efficiency.

**Table 2:** Ablation experimental results of YOLO-SD model.

Model	DySample	SPD-Conv	Precision(%)	Recall(%)	mAP50(%)	mAP50-95(%)	Parameters(M)	FLOPs(G)	Model size(MB)
YOLO11n-seg	—	—	88.7	89	94.0	85.1	2.83	10.2	6.1
Model1	√	—	90.3	90.1	95.0	86.4	2.84	10.2	6.2
Model2	—	√	91.2	88.6	95.0	85.7	2.46	9.3	5.4
YOLO-SD	√	√	90.5	90.3	95.2	86.2	2.47	9.3	5.4

#### 3.3.2 Comparison experiment

To verify the effectiveness of the proposed model in apple instance segmentation tasks, we conducted comparative experiments between the classic instance segmentation model Mask RCNN (He et al., 2017) and the YOLO series mainstream segmentation model with the improved model (**Table 3**).

**Table 3:** Comparison experimental results of YOLO-SD model.

Model	Precision(%)	Recall(%)	mAP50(%)	Parameters(M)	FLOPs(G)	Model size(MB)
YOLO8n-seg	89.9	88.3	93.9	3.26	12.1	6.9
YOLO11n-seg	88.7	89	94.0	2.83	10.2	6.1
YOLO12n-seg	86.8	85.3	91.9	2.76	9.7	6.1
Mask-RCNN	76.8	78.1	80.8	28.00	/	115.2
YOLO-SD	90.5	90.3	95.2	2.47	9.3	5.4

Observed from the experiment results in **Figure 10**; **Table 3**, the YOLO-SD model proposed demonstrated the optimal comprehensive performance in the apple instance segmentation task. In terms of segmentation performance, YOLO-SD outperformed the mainstream comparison models by 95.2% in mAP50. Specifically, its mAP50 value is 1.2 percentage points higher than the second-best YOLO11n-seg and 15.1 percentage points higher than the classic Mask R-CNN. In terms of model efficiency, YOLO-SD only requires 2.47M parameters and 5.4MB storage space, with a computational complexity of 9.3 GFLOPs, which is the lowest among the compared models. In comparison with models in the same series, YOLO-SD significantly improved segmentation accuracy while reducing parameter quantity by 24.2%, 12.7%, and 10.5% compared to YOLOv8n-seg, YOLO11n-seg, and YOLO12n-seg, respectively. The model volume also decreased by 21.7%, 11.5%, and 11.5%, achieving effective control of model complexity while maintaining high accuracy.

To verify whether the performance improvement of the improved model has statistical reliability, this study used paired sample t-test to test the differences between the YOLO-SD model and the baseline model. The results indicate that the t-statistic is 4.8314, corresponding to a p-value of 0.0085, which is much lower than the preset significance level α=0.05. The performance mean of the improved model is significantly better than that of the baseline model.

#### 3.3.3 Recognition results of the YOLO-SD model for apples with different growth states and occlusion conditions

The YOLO-SD model has excellent detection performance for fruits under different growth states and occlusion conditions, with the mAP50 reaching 95.2% and mAP50–95 reaching 86.2% (**Table 4**). Among them, the model has the best detection performance for categories with less occlusion and obvious features (e.g., ‘single-apple-none’, ‘multiple-front-none’). They achieved an average accuracy of 98.0% and 97.3% respectively, demonstrating the high accuracy of the model in ideal scenarios. For categories with occlusion caused by foliage or other objects, the model still maintained excellent performance. Among them, the mAP50 value of ‘single-apple-leaf’ reached 95.7%, and that of ‘multiple-front-none’ reached 95.4%, demonstrating that the introduced module effectively strengthens the model’s capability to extract features and localize partially occluded targets. Moreover, when the model dealt with complex scenes with multiple rear parts and occlusions, the mAP50 value still exceeded 92%, demonstrating the model maintains a high detection accuracy in complex backgrounds.

**Table 4:** Recognition results of the YOLO-SD model for apples with different growth states and occlusion conditions.

Category	Precision(%)	Recall(%)	mAP50(%)	mAP50-95(%)
single-apple-none	92.8	93.9	98	96.3
single-apple-leaf	91.0	91.4	95.7	87.8
single-apple-other	88.5	88.8	94.8	85.5
multiple-front-none	92.3	95.0	97.3	95.2
multiple-front-leaf	89.9	91.4	95.4	89.5
multiple-front-other	88.9	87.2	93.5	85.3
multiple-back-none	90.8	93.9	96.5	88.8
multiple-back-leaf	88.9	85.2	92.8	79.2
multiple-back-other	91.6	86.0	93.2	78.6
Average	90.5	90.3	95.2	86.2

### 3.4 Keypoint detection results and analysis of canopy apples

#### 3.4.1 Ablation experiment

This study conducted performance analyses on each of the introduced modules through ablation experiments. As shown in **Table 5**, every module has a positive influence on the model’s performance. After replacing the original convolutional layer with the RFAConv module, the model’s precision, recall rate, and mAP50 values have respectively increased by 0.6%, 0.3%, and 0.8%, which proves that this module enhances the local feature perception ability, effectively improving the model’s recognition accuracy for occluded small targets. However, the parameter quantity and computational cost have increased at the same time. After introducing the SCSA attention mechanism, the mAP50 metric increased by 0.5 percentage points and maintained the computational cost and model parameters basically unchanged meanwhile, indicating the module can enhance model’s focus ability on key areas with extremely low resource consumption and has excellent computational efficiency. By adopting the MBConv structure, the parameter and computational complexity were respectively decreased by 12.4% and 19.0%, effectively compressing the model size while maintaining benchmark accuracy. Ultimately, the YOLO-RSM model outperformed the baseline model by 2.1% in precision, 1.7% in recall, and 2.6% in mAP50, while the parameters and computational cost have only increased by 3.9% and 12.7%. This achieved a significant improvement in overall performance under limited computational resources, demonstrating the effectiveness of collaborative optimization among modules.

**Table 5:** Ablation experimental results of YOLO-RSM model.

Model	RFA-Conv	SCSA	MBConv	Precision (%)	Recall (%)	mAP50 (%)	Parameters (M)	FLOPs (G)	Model size(MB)
YOLO11n	—	—	—	86.9	85.5	87.7	2.58	6.3	5.6
Model1	√	—	—	87.5	85.8	88.5	3.00	8.4	6.4
Model2	—	√	—	87.2	85.0	88.2	2.58	6.3	5.6
Model3	—	—	√	85.7	86.3	87.6	2.26	5.1	5.0
YOLO-RSM	√	√	√	89	87.2	90.3	2.68	7.1	5.8

#### 3.4.2 Comparison experiment

The YOLO-RSM algorithm proposed in this article was compared with mainstream object detection models such as YOLOv8n, YOLO11n, YOLO12n and RT-DETR. As is presented in **Table 6**, the precision, recall rate, mAP value of YOLO-RSM reached 89.0%, 87.2% and 90.3% respectively. Compared with the mainstream model YOLOv8n, YOLO-RSM has achieved an increase of 3.4 percentage points in mAP50 while reducing the computational complexity. Meanwhile, compared with the same magnitude model YOLO11n and YOLO12n, YOLO-RSM maintained a relatively stable computational load while achieving an increase of 2.6% and 4.7% in mAP50 respectively. Compared with RT-DETR which adopts the Transformer architecture, the mAP50 of YOLO-RSM is 1.4 percentage points higher. Moreover, the parameter quantity and model size of YOLO-RSM is less than one-tenth of that of RT-DETR. This fully demonstrates that YOLO-RSM not only maintains high detection accuracy but also has excellent lightweighting and deployment advantages. Furthermore, the mAP curve of the training period shown in **Figure 11** indicates that this model consistently maintains a leading performance, demonstrating superior convergence and feature extraction capabilities.

**Table 6:** Comparison experimental results of YOLO-RSM model.

Model	Precision (%)	Recall (%)	mAP50 (%)	Parameters (M)	FLOPs (G)	Model size (MB)
YOLOv8n	86.1	86.0	86.9	3.01	8.1	6.4
YOLO11n	86.9	85.5	87.7	2.58	6.3	5.6
YOLO12n	83.2	84.7	85.6	2.50	5.8	5.6
RT-DETR	89.5	87.0	88.9	33.25	88	67.3
YOLO-RSM	89.0	87.2	90.3	2.68	7.1	5.8

Similarly, we used t-test to test the difference between YOLO-RSM model and baseline model. The results showed that the t-statistic was 2.9268, with a corresponding p-value of 0.0429, indicating that at a significance level of 0.05, the improved model performed significantly better than the baseline model in keypoint recognition tasks.

**Figure 12:**
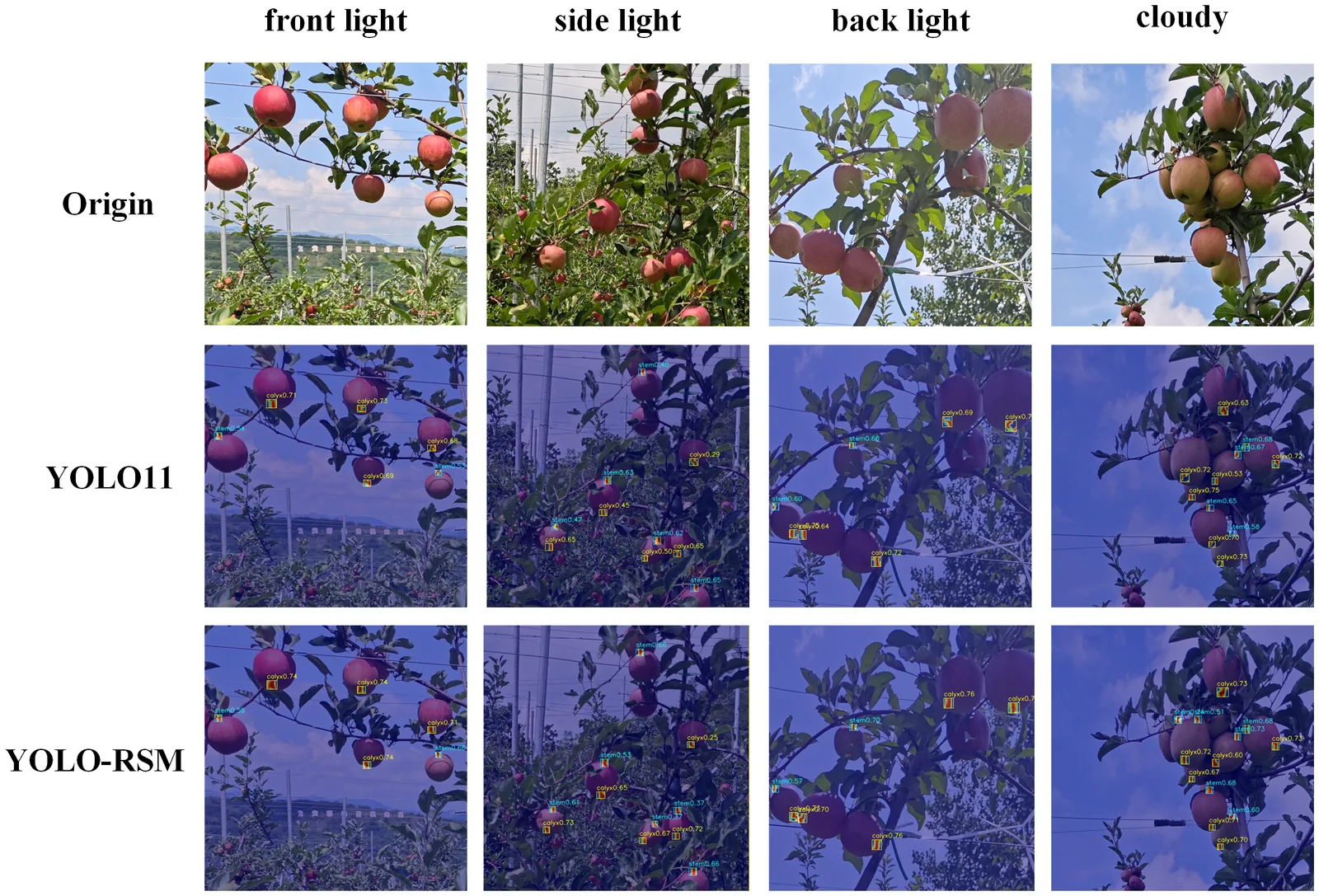
Visual heatmap of keypoint identification.

By comparing the heatmap visualization results of the stem and calyx region (**Figure 12**), we can observe that the original YOLO11 model has a relatively scattered attention distribution when detecting the keypoints of the stem and calyx, making it difficult to accurately target the fine-grained features. The activation areas are often disturbed by the surface texture of the fruit, as well as the surrounding branches and leaves and other background information. This results in insufficient extraction of keypoint features, making it prone to localization errors or missed detections. On the contrary, the heat map of YOLO-RSM model exhibits stronger feature focusing ability. The high-response areas precisely concentrate on the stems and calyxes, and the boundaries are clearly convergent, proving that the improved algorithm can significantly improve the model’s recognition accuracy and anti-interference capability for keypoints.

### 3.5 2D axis estimation results

Considering the practical needs of robot harvesting operations, fruits in the scenes of single and front of multiple fruits are regarded as the main operation targets due to their ease of harvesting, and a certain degree of leaf obstruction does not affect lossless grasping. Therefore, this study conducted classification evaluation and index calculation for the four types of apple targets that are suitable for harvesting, and further evaluated the accuracy rate of their axis estimation. The specific results are listed in **Table 7**.

**Table 7:** The keypoint recognition accuracy of harvestable apples.

Category	Precision (%)		Recall (%)		mAP50(%)	
	Stem	Calyx	Stem	Calyx	Stem	Calyx
single-apple-none	90.2	89.8	90.8	91.1	91.6	91.7
single-apple-leaf	91.9	87.7	87.6	85.6	92.5	87.1
multiple-front-none	86.3	94.0	85.9	94.8	86.6	94.4
multiple-front-leaf	85.7	92.2	82.2	85.4	84.7	88.2
Average	88.52	90.92	86.63	89.23	88.85	90.35

In the scenario of the single fruits without obstruction, background interference is minimized, thus the model is able to make full use of the local features of keypoints. The average accuracy of keypoint recognition for stems and calyxes reaches 91.6% and 91.7% respectively, and the accuracy and recall rates are also maintained at around 90%. In the scenario of single fruits with leaf obstruction, the accuracy of stem recognition remains at a high level, with mAP50 reaching 92.5%, while various indicators of calyx recognition decrease by 4%-6% compared to the unobstructed scenario. In the unobstructed scene of the front part of multiple fruits, the model has the most outstanding detection effect on the calyx, with mAP50 increasing to 94.4%, significantly outperforming the detection effect on the calyx in the same scene. In the case of the front part of multiple fruits superposing leaf obstruction, the recognition performance of stem and calyx key points showed a certain degree of decline, with the mAP50 of calyx decreasing to 88.2% and the mAP50 of stem decreasing to 84.7%.

To quantitatively evaluate the model’s prediction performance on the 2D axis of apples, this study used Labelme software to manually label the 2D axis of apples. A total of 509 apple samples were labeled. For samples where both keypoints were visible, the axis vector was from the calyx to the fruit stalk. For samples where only one keypoint was visible, the axis vector was from the center point fitted by the algorithm to the stem (only the stem was visible) or from the calyx to the center point (only the calyx was visible). Additionally, a total of 96 samples with both keypoints being invisible were identified, accounting for 15.9% of the total samples. Due to the complex environment of the orchard, these samples mainly occurred because the fruits were severely obscured, resulting in both keypoints being invisible. On such samples, neither manual nor algorithmic methods could provide reliable axis true values. Therefore, we did not consider these apples when calculating the axis estimation accuracy rate.

**Figure 13:**
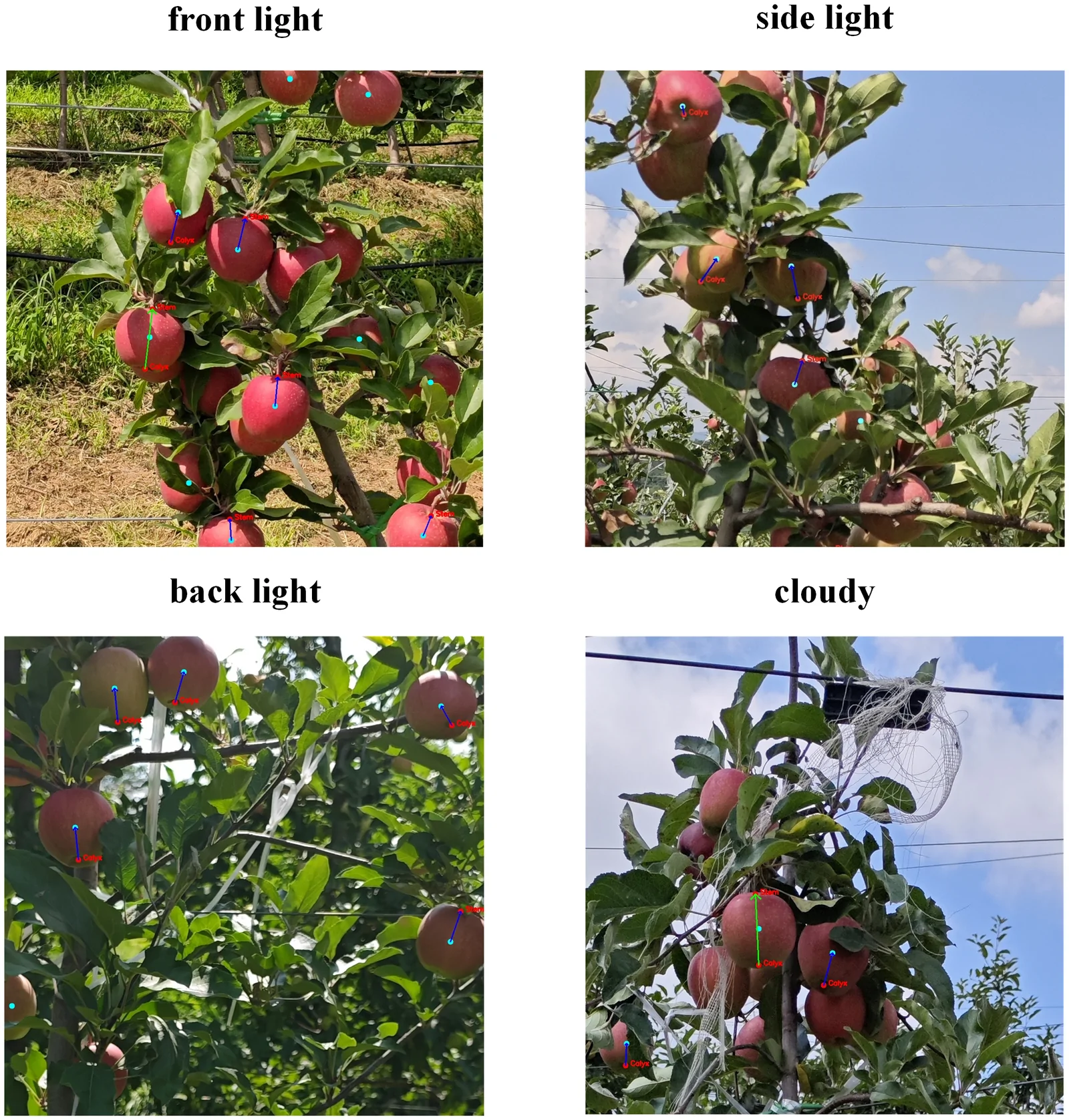
Axis estimation results. The green arrow represents the axis of the apple fruit where both keypoints are visible and the blue arrow represents the axis of the apple fruit where only one key point is visible.

To verify the statistical robustness of the algorithm, this study conducted three repeated training sessions on the instance segmentation and keypoint detection models using random seeds 0, 50, and 100, and calculated the results of the axis estimation. The experimental results show that the mean angle error (MAE) obtained by the proposed method is 7.23° ± 16.73°. To further analyze the statistical distribution characteristics of the error, the quantile indicators were introduced for analysis. The results indicate that the median of the angle error is only 2.31°, the lower quartile (Q1) and the upper quartile (Q3) are 1.08° and 4.51° respectively, and 90% of the samples (P90) have an angle error within 10.26°, indicating that the algorithm can achieve relatively accurate axis estimation in most cases. However, due to the misidentification and omission of keypoints, the maximum error is 178.53°, usually caused by the misidentification of keypoints due to occlusion, resulting in a direction reversal. When using the angle error *θ* ≤ 15° as the judgment threshold, the axis estimation accuracy (AEA) is 92.68% ± 1.22%, indicating that the proposed method has high reliability in actual picking operations. **Figure 13** presents the axis estimation results under different lighting conditions.

Furthermore, in order to comprehensively evaluate the deployment adaptability of this method on edge devices, this study deployed the trained cascaded axial recognition model on a mobile edge computing device equipped with NVIDIA GeForce RTX 4060 Laptop graphics card for testing, and compared the results with those on the host device. The experimental results show that the inference latency of this algorithm on the host device is 62.67 ms, achieving a real-time throughput of 15.96 FPS. On the edge device, the inference latency is 137.87 ms, achieving a real-time throughput of 7.25 FPS. This can meet the response requirements for real-time detection by the robot and real-time picking control by the mechanical arm. It indicates that this method still maintains efficient axis recognition performance when deployed on edge devices.

### 3.6 External dataset validation

In this study, the algorithm was verified using the external dataset consisting of Gala and Fuji apples collected by the Intel Realsense D456 depth camera in Luoning County, Luoyang City on August 29, September 20, and September 24, 2025. The experimental results in **Table 8** showed that the average detection accuracy mAP of the YOLO-SD model for four types of harvestable apples reached 88%. Among them, the mAP values for the four categories of “single-apple-none”, “single-apple-leaf”, “multiple-front-none”, and “multiple-front-leaf” reached 91.9%, 88.3%, 87.6%, and 84.2% respectively. The mAP50 of this model has increased by 2.2 percentage points compared to the baseline model YOLO11-seg, and has increased by 3.6 and 5.9 percentage points respectively compared to YOLOv8-seg and YOLOv12-seg. The overall mAP of the YOLO-RSM model reached 83.4%, with the recognition accuracy for the fruit stem being 81.1% and for the calyx being 85.6%. The YOLO-RSM model outperforms the baseline model YOLO11 by 4.2 percentage points, and it also improves by 2.8 and 5.2 percentage points compared to YOLOv8 and YOLOv12 respectively. Compared with the original dataset, the performance of the model on the external validation set has declined. The main reason for this is that the resolution of the depth camera images can only reach 1280×720, which prevents the full expression of the edge features of apple instances and the keypoints of small targets. This limitation stems from the inherent physical characteristics of the depth sensor and is quite common in depth cameras. Moreover, the external dataset contains some Gala apples, which have certain differences in morphology from Fuji apples, thereby affecting the performance of the model. In the future, by introducing targeted hyperparameter optimization strategies or fine-tuning mechanisms, the detection accuracy of the model on low-resolution external datasets can be further improved.

**Table 8:** Results of external dataset comparison tests.

Model	Instance segmentation			Model	Keypoint detection		
	Precision(%)	Recall(%)	mAP50(%)		Precision(%)	Recall(%)	mAP50(%)
YOLOv8-seg	82.1	79.6	84.4	YOLOv8	83.4	77.1	80.6
YOLO11-seg	80.9	81.5	85.8	YOLO11	78.7	81.4	79.2
YOLOv12-seg	83.4	79.3	82.1	YOLOv12	79.6	75.9	78.2
YOLO-SD	86.3	85.2	88.0	YOLO-RSM	86.7	80.0	83.4

## 4 Discussion

### 4.1 Analysis of false negative and false positive cases

**Figure 14:**
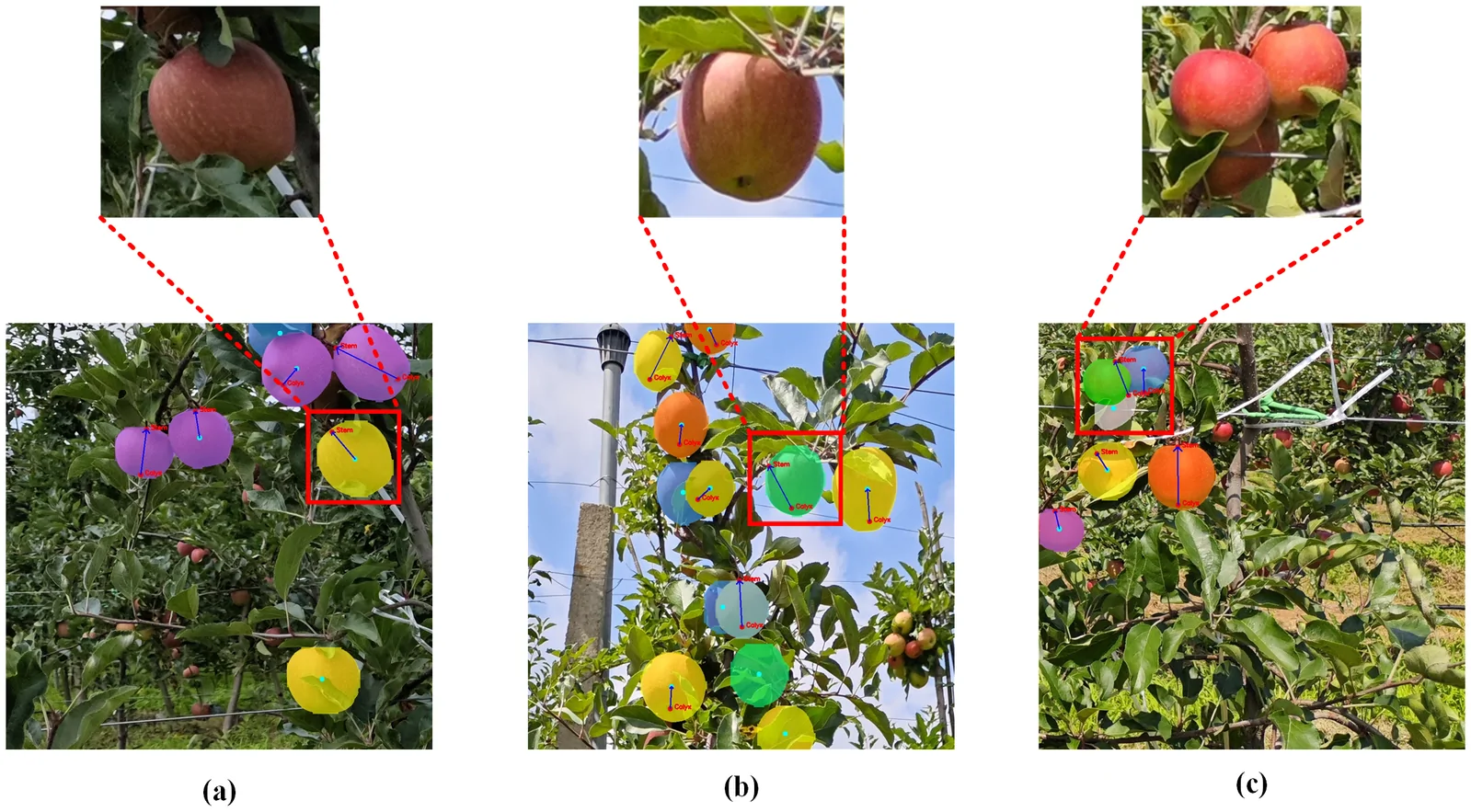
False Negative (FN) and False Positive (FP) cases. **(a)** FN caused by weak light and local occlusion, **(b)** FP caused by the interference of tree branches, **(c)** FP and FN caused by texture similarity and semantic ambiguity in multi-fruit clusters.

Through qualitative analysis of the experimental results in **Figure 14**, it was found that although the improved model performed well in most scenarios, there were still error cases in some extreme lighting and severe occlusion conditions. The fruits in **Figure 14a** and the fruits on the right side of **Figure 14c**, the calyxs and stems of the fruits were partially obstructed by the leaves and located in low-light shadow area, which resulted in significant weakening of the texture and edge features in keypoint areas. The model failed to effectively distinguish foreground targets from background noise during feature extraction, and mistakenly suppressed the calyx and stem regions as background, thereby resulting in missed detection of keypoints. In **Figure 14b**, the main part of the fruit stalk was obscured by the leaves, while the adjacent area of the fruit contains branches that were extremely similar in shape to the fruit stem. Due to the high geometric resemblance in color, diameter and extension direction between branches and fruit stems, the model failed to correctly establish the topological relationship between the fruit body and keypoints in the lack of target features in the absence of target features, and mistakenly regarded the branches in the background as the fruit stems. In the fruit on the left side of **Figure 14c**, its calyx was completely obscured by leaves, resulting in a complete loss of visual information. The dark shadows formed by the clustered areas of multiple fruits have a high degree of visual isomorphism with the calyxs in terms of morphology and gray-scale distribution. The model failed to integrate global contextual information and incorrectly inferred the shadows at the intersection of multiple fruits as calyx.

### 4.2 Advantages

In this study, a complete computational framework that encompasses instance segmentation, keypoint detection, and 2D pose estimation has been established. This method adopts a cascaded strategy that combines instance screening with key point detection. Through the detailed instance segmentation of nine categories, pickable apples are selected. A dynamic picking target queue is then constructed based on semantic logic, and serves for the subsequent planning of picking sequence. Kok and Chen (2024) adopted a two-stage heterogeneous model architecture based on DaSNet-v2 and HigherHRNet for instance segmentation and keypoint detection, achieving a median angle error of 17.6° on public datasets, whereas this paper’s median angle error is only 2.05°. Additionally, the architecture utilized multiple independent models in series, introducing significant computational and communication overheads. In contrast, this paper uses the YOLO model in both stages, avoiding the additional costs caused by multi model switching, data transmission, and format conversion. Furthermore, the single-stage multi-task model proposed by Zhao et al. (2025) unifiedly regresses the entire image for multiple tasks, achieving a segmentation accuracy of 93.8% and a keypoint recognition accuracy of 88.2%. However, in cases of severe occlusion, when the two tasks share the same feature extraction backbone, it may lead to feature conflicts. In contrast, the two-stage design of this paper decouples instance segmentation and keypoint detection in terms of space and tasks, allowing each sub-task to focus on its own optimal goal. This resulted in a segmentation accuracy of 95.1% and a keypoint recognition accuracy of 90.2%. Moreover, through the screening in the first stage, the qualified targets were selected to enter the second-stage keypoint prediction, significantly reducing redundant computations.

Furthermore, in terms of model improvement, this paper has proposed targeted model improvement methods based on the characteristics of different sub-tasks. On the one hand, to enhance the recognition effect and model efficiency of apples with different degrees of occlusion, a lightweight instance segmentation model YOLO-SD was proposed. Compared with the apple detection and localization method using the improved BlendMask proposed by Bai et al. (2024), the YOLO-SD model achieves fine classification of nine types of occlusion scenarios while maintaining high accuracy. It only requires 2.47 M parameters and 9.3 GFLOPs of computational cost, achieving a better balance between accuracy and lightweighting. On the other hand, to solve the challenges of detecting small-scale keypoints, a keypoint detection model YOLO-RSM was designed that integrates receptive field attention, spatial channel collaborative attention, and inverted bottleneck structure. The architecture greatly improves the model’s capability to capture and discriminate small target features.

### 4.3 Error propagation analysis

For the two-stage model, the errors in the first stage will inevitably propagate to the second stage. In the instance segmentation stage, there are two types of errors: one is false positive (FP), where non-apple areas are wrongly identified as apples. This type of error will be naturally filtered out in the second stage because the subsequent keypoint detection model will independently evaluate the input ROI area and will not calculate the axes of valid keypoints if no effective keypoints are detected. The other is false negative (FN), where real apples are missed. This type of error does indeed prevent the target from entering the second stage, but such missed detections are not a problem specific to the cascaded structure; single-stage models also face missed detection issues. In **Table 4**, the segmentation accuracy of the four types of harvestable apples reached 98%, 95.7%, 97.3%, and 95.4% respectively. This indicates that the vast majority of harvestable apples were correctly segmented and passed to the second stage, thereby minimizing the risk of error propagation. Therefore, the FP errors in the first stage will be suppressed in the second stage, while FN errors will exist in both single-stage and multi-stage models. Thus, the error propagation of the cascaded architecture in this paper is actually gradually attenuated. In the application of the picking robot, mis-detection will cause the mechanical arm to collide with the fruit, while missed detection can be remedied by re-picking after eliminating the occlusion in the subsequent stage. Therefore, the cascaded architecture is reasonable in the picking task.

### 4.4 Limitations and future work

Though the two-stage axis estimation strategy proposed in this study efficiently addresses the ambiguity problem in keypoint attribution in multi-fruit clustering scenarios and obtains the 2D axis direction of the apples by independently inputting pickable apple instances into the keypoint detection network, and significantly improves the detection accuracy of subtle features, this method still faces certain limitations in practical applications. According to the analysis of error cases in section 4.1, the model still lacks the ability to accurately calculate the axis direction of the apple when it encounters scenarios with dense occlusion and the keypoints of the target are completely covered. In addition, the current algorithm calculates the axial information of apples based on 2D images. However, the 2D axis information is unable to fully reflect the actual posture and direction information of apples in 3D space. In response to the above limitations, future research work can focus on the following two directions: One approach is to combine physical intervention methods, such as flexible flap mechanisms or directional airflow devices, to actively remove or blow away obstructing leaves before picking, thereby eliminating the obstruction and obtaining the keypoint position information. This enhances the recognition accuracy of the model in extreme obstruction scenarios and improves the overall usability of the system. Additionally, another approach is to transfer the algorithm to the dataset obtained from depth cameras. We can use the depth information and the corresponding RGB images to perform alignment operations to obtain a 3D point cloud in the spatial domain. After the algorithm segments the apple instances and detects the 2D keypoints, it uses the instance segmentation mask to filter out the background point cloud, and only retain the fruit point cloud of a single apple. At the same time, the depth values are mapped to the corresponding pixel positions to obtain the 3D coordinates of the keypoints in the camera coordinate system. Then, the spatial position of the center point is fitted in the 3D space. Subsequently, the 2D axis vector is projected onto the 3D point cloud space, and the true axis direction of the apple in the 3D space is calculated.

## 5 Conclusion

To solve the issues of apple instance segmentation being susceptible to occlusion interference and insufficient recognition accuracy of small-scale keypoints in complicated orchard environments, this study proposes two improved models. Firstly, a lightweight YOLO-SD instance segmentation model is constructed, achieving accurate segmentation of different categories of apples in the canopy. Secondly, a YOLO-RSM keypoint detection model is developed, strengthening the model’s capability to distinguish subtle features of keypoints. Finally, a method for estimating the 2D axis orientation of apples is proposed based on the optimized YOLO models above. The experimental results show that the YOLO-SD model achieved an mAP50 of 95.2% and an mAP50–95 of 86.2% on the apple test set with nine different growth states and occlusion conditions, and the parameter size and model size were only 2.47 M and 5.4 MB respectively. The precision rate, recall rate and average accuracy of the YOLO-RSM model have reached 89.0%, 87.2% and 90.3% respectively. It achieved an mAP50 of 90.35% (for the calyx) and 88.85% (for the stem) on four types of edible apples at the same time. The average angular error (MAE) was only 7.23° ± 16.73°, and the axis estimation accuracy have reached 92.68%. This study provides a feasible solution for harvesting robots to synchronously achieve precise fruit segmentation and robust detection of attitude keypoints in complex occlusion scenes, which has positive significance for promoting the practical application of automated apple harvesting.

## Data Availability

The raw data supporting the conclusions of this article will be made available by the authors, without undue reservation.
